# Inhibition of intestinal FXR activity as a possible mechanism for the beneficial effects of a probiotic mix supplementation on lipid metabolism alterations and weight gain in mice fed a high fat diet

**DOI:** 10.1080/19490976.2023.2281015

**Published:** 2023-11-20

**Authors:** Alice Beau, Bérengère Benoit, Mélanie Le Barz, Emmanuelle Meugnier, Armelle Penhoat, Catherine Calzada, Claudie Pinteur, Emmanuelle Loizon, Stéphanie Chanon, Aurélie Vieille-Marchiset, Valérie Sauvinet, Murielle Godet, Fabienne Laugerette, Sophie Holowacz, Elsa Jacouton, Marie-Caroline Michalski, Hubert Vidal

**Affiliations:** a Laboratoire CarMeN, INSERM U.1060, INRAe U. 1397, Université Claude Bernard Lyon1, Pierre Bénite, France; b Centre de Recherche en Nutrition Humaine - Rhône-Alpes, INSERM, INRAe, Université Claude Bernard Lyon1, Hospices Civils de Lyon, Pierre Bénite, France; c Research & Development Department, PiLeJe Laboratoire, Paris, France

**Keywords:** Metabolic syndrome, high-fat diet, bifidobacterium animalis, lactobacillus gasseri, gut microbiota, bile acids, intestinal FXR signaling, lipid metabolism

## Abstract

Supplementation with probiotics has emerged as a promising therapeutic tool to manage metabolic diseases. We investigated the effects of a mix of *Bifidobacterium animalis* subsp. *lactis* LA804 and *Lactobacillus gasseri* LA806 on high-fat (HF) diet -induced metabolic disease in mice. Supplementation with the probiotic mix in HF diet-fed mice (HF-Pr2) reduced weight and fat mass gains, decreased hepatic lipid accumulation, and lowered plasma triglyceride peak during an oral lipid tolerance test. At the molecular level, the probiotic mix protected against HF-induced rise in mRNA levels of genes related to lipid uptake, metabolism, and storage in the liver and white adipose tissues, and strongly decreased mRNA levels of genes related to inflammation in the white adipose tissue and to oxidative stress in the liver. Regarding intestinal homeostasis, the probiotic mix did not prevent HF-induced gut permeability but slightly modified microbiota composition without correcting the dysbiosis induced by the HF diet. Probiotic supplementation also modified the cecal bile acid (BA) profile, leading to an increase in the Farnesoid-X-Receptor (FXR) antagonist/agonist ratio between BA species. In agreement, HF-Pr2 mice exhibited a strong inhibition of FXR signaling pathway in the ileum, which was associated with lipid metabolism protection. This is consistent with recent reports proposing that inhibition of intestinal FXR activity could be a potent mechanism to overcome metabolic disorders. Altogether, our results demonstrate that the probiotic mix evaluated, when administered preventively to HF diet-fed mice could limit obesity and associated lipid metabolism disorders, likely through the inhibition of FXR signaling in the intestinal tract.

## Introduction

Energy imbalance due to unhealthy eating habits and low physical activity contributes to the dramatic rise in the prevalence of chronic metabolic diseases and associated cardiovascular risk.^1–3^ More than one billion people worldwide are presently suffering from obesity and/or metabolic syndrome (MetS), defined as a pathological state characterized by at least three of the following conditions: abdominal obesity, insulin resistance, hypertension, and hyperlipidemia.^4^ This cluster of conditions increases the risk of heart diseases, stroke, nonalcoholic fatty liver disease (NAFLD), and type 2 diabetes.^1,4^ Facing the severity of these pathologies as well as their considerable economic burden for public health systems, new therapeutic but also preventive strategies are needed.

Obesity and MetS have been associated with gut microbiota alterations and disruption of intestinal homeostasis with an impact on nutrient absorption, inflammation, intestinal barrier functions, gut hormone secretion, and production of important vitamins and metabolites.^5–7^ The gut microbiota is generally altered, both in terms of richness and diversity, in situations of obesity and metabolic diseases in rodent models as well as in humans.^6,7^ Furthermore, the use of germ-free animals and experiments of fecal transplantation from obese donors (mice or humans) to recipient lean mice support a causal role of intestinal bacteria in the development and maintenance of these pathologies.^8–10^ However, the precise mechanisms linking the changes in gut microbiota and host physiology are still largely unraveled because several processes could occur in synergy or independently. These include the production of short chain fatty acids (SCFAs), the production or modulation of a variety of bioactive molecules (indoles, bile acids, polar lipids, vitamins, amino acids, etc.), regulation of the intestinal mucus layer, intestinal permeability, as well as inflammation and immunity.^5–7,11–13^ These effects could also vary according to location in the intestinal tract. Consequently, targeting the gut microbiota to fight obesity and MetS is an important avenue of research, and the use of probiotics to restore intestinal homeostasis has appeared over the years as one of the promising strategies for this aim.

A recent systematic review of randomized clinical trials testing lactic acid bacteria has identified 23 studies reporting significant weight loss.^14^ Some reports have also shown the effects of probiotics on biomarkers associated with MetS. In a randomized trial, administration of a mixture of four probiotic strains to obese children was associated with a decrease in liver markers (ALAT and ASAT), cholesterol, low-density lipoprotein-cholesterol, and triglyceride (TG) levels, as well as a decrease in waist circumference.^15^ The use of another multi-strain probiotic mix improved levels of ALAT, cytokines, and endotoxins, as well as liver histology in adult patients with NAFLD.^16^ The consumption of *Lactilactobacillus curvatus* HY7601 and *Lactiplantibacillus plantarum* KY1032 by non-diabetic patients with hypertriglyceridemia also reduced lipid catabolism and lipoprotein lipase activity, hence lowering plasma TG levels.^17^ There is therefore evidence of efficacy that supplementation with probiotics could be useful for the management and potential prevention of MetS and related pathologies^18^. However, additional work is clearly needed to better define the mechanisms of action of promising probiotics in pre-clinical models prior to clinical evaluation. Recently, screening of candidates identified several strains with potential for the treatment or prevention of obesity and MetS.^19,20^ Beneficial effects were generally observed when different strains were administered simultaneously such as *Lacticaseibacillus rhamnosus* Lb12 and *Bifidobacterium animalis* subsp. *lactis* Bf141, which were able to improve the integrity of the intestinal barrier,^19^ or *B. animalis* subsp. *lactis* LA804 and *Lactobacillus gasseri* LA806, which were able to reduce body weight gain and inflammation^20^ in mice fed a high-fat (HF) diet.

The objective of this study was to characterize in detail the beneficial effects of the combination of the two probiotic strains *B. animalis* subsp. *lactis* LA804 and *Lactobacillus gasseri* LA806,^20^ and to gain insight into their mechanisms of action in preventing metabolic disorders in a mouse model of obesity and MetS induced by the HF diet. Markers of lipid metabolism, inflammation, and oxidative stress, as proxies of MetS, were specifically assessed. A thorough evaluation of intestinal homeostasis was also performed to gain a better understanding of the mechanisms of action of the probiotic mix.

## Results

### Probiotic mix supplementation reduced weight gain, fat accumulation, and hyperinsulinemia in HF diet-fed mice

**Figure 1.**
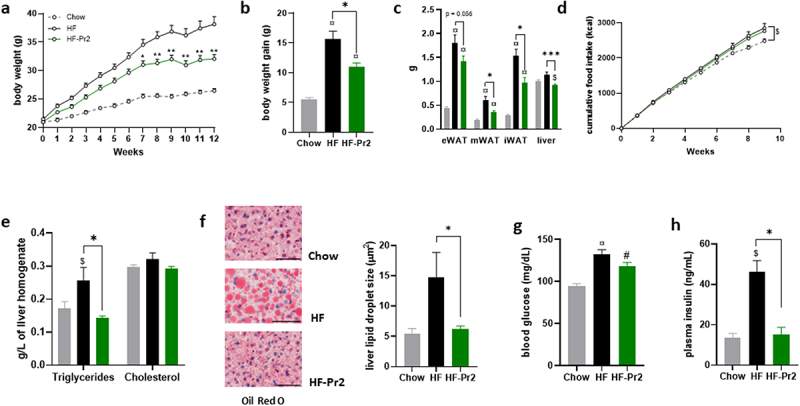
Supplementation with the probiotic mix attenuates weight gain and fat mass accumulation and metabolic alterations in HF diet-fed mice. (a) body weight evolution (*n* = 12–16). (b) body weight gain at 12 weeks. (c) epididymal white adipose tissue (eWAT), mesenteric white adipose tissue (mWAT), inguinal white adipose tissue (iWAT) and liver weights at sacrifice. (d cumulative food intake recorded during 10 weeks. (e) liver triglycerides and total cholesterol. (f) oil-red O liver sections and liver lipid droplet size (*n* = 6–12); 60× magnification; bar = 25 µm. (g) fasting blood glucose level (*n* = 15–16). (h) fasting plasma insulin level (*n* = 5). Data are mean ± SEM. ^$^*p <0.05*, ^#^*p <0.01*, ^¤^*p <0.001 versus* the Chow diet group and *p <0.05, **p <0.01, ***p <0.001 for the HF-Pr2 *versus* the HF group.

Mice were fed a HF diet for 12 weeks. The probiotic mix was administered daily from the first day of the HF diet until the end of the experiment. Compared to Chow diet fed mice, HF diet-fed mice showed greater body weight gain (Figure 1a,b) and significantly higher weights of the three white adipose tissue deposits (Figure 1c). Supplementation with the probiotic mix significantly protected against the effects of the HF diet on body weight gain (Figure 1a,b) and adipose tissue accumulation in all fat depots (Figure 1c). Importantly, the probiotic mix did not influence food intake (Figure 1d). The HF diet also increased the amount of TG and the size of lipid droplets accumulated in the liver (Figure 1e,f) whereas probiotic mix intake counteracted these effects (Figure 1e,f) and reduced liver weight (Figure 1c). Hepatic cholesterol levels were not modified in any of the groups (Figure 1e).

Six-hour fasting plasma TG, cholesterol, glucose, and insulin concentrations were measured at the end of the intervention. TG and total cholesterol levels were significantly increased in the HF diet-fed mice compared to the Chow diet fed group, but the probiotic mix treatment did not affect these values TG: 0.90 ± 0.06 mmol/L (HF), 1.16 ± 0.09 mmol/L (HF-Pr2), and 0.72 ± 0.04 mmol/L (Chow), *p* = 0.0003 Chow *vs* HF-Pr2. Total cholesterol: 7.75 ± 0.11 mmol/L (HF), 7.15 ± 0.36 mmol/L (HF-Pr2) and 5.43 ± 0.18 mmol/L (Chow), *p* = 0.001 HF *vs* Chow, *p* = 0.002 HF-Pr2 *vs* Chow). In the HF-fed group, glycemia and insulinemia were significantly increased compared to the Chow diet fed group (Figure 1g,h), indicative of a state of insulin resistance and glucose intolerance. Supplementation with the probiotic mix did not fully restore fasting glycemia but maintained fasting insulin concentrations at the level found in Chow diet fed mice (Figure 1g,h).

### Probiotic mix supplementation was associated with reduced expression of lipid storage genes and of oxidative stress and inflammatory markers in liver and adipose tissue

**Figure 2.**
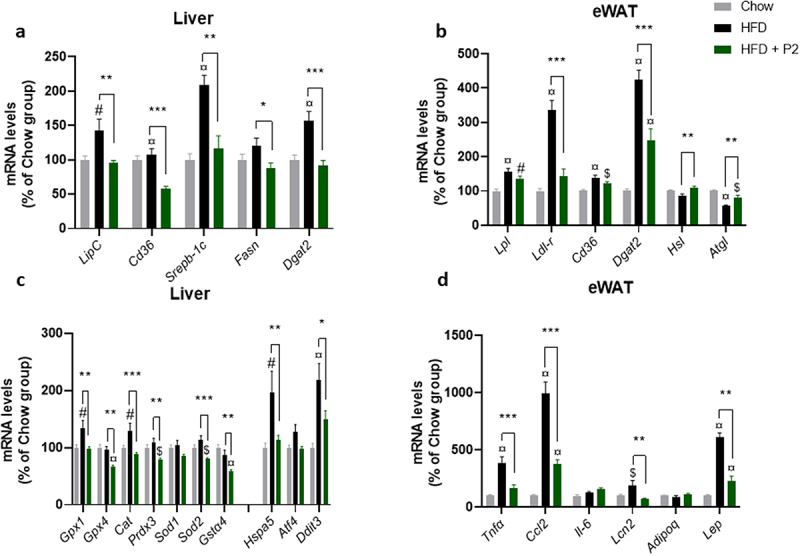
Effects of the probiotic mix on gene expression in the liver and eWAT. (a) relative mRNA expression levels of lipid metabolism-related genes in the liver: hepatic lipase (*LipC*), CD36 antigen (*CD36*), sterol regulatory element binding transcription factor 1 (*Srebp-1c*), fatty acid synthase (*Fasn*), and diacylglycerol O-acyltransferase 2 (*Dgat2*) (*n* = 7–10). (b) relative mRNA expression levels of lipid metabolism-related genes in eWAT: lipoprotein lipase (*Lpl*), low density lipoprotein receptor (*Ldl-r*), CD36 antigen (*CD36*), diacylglycerol O-acyltransferase 2 (*Dgat2*), hormone sensitive lipase (*Hsl*) and adipose triglyceride lipase (*Atgl*) (*n* = 5–8). (c) relative mRNA expression levels of oxidative stress and ER stress-related genes in the liver: glutathione peroxidase 1 (*Gpx1*) and 4 (*Gpx4*), Catalase (*Cat*), peroxiredoxin 3 (*Prdx3*), superoxide dismutase 1 (*Sod1*) and 2 (*Sod2*), glutathione S-transferase alpha 4 (*Gsta4*), heat shock 70kDa protein 5 (*Hspa5*), activating transcription factor 4 (*Atf4*) and DNA-damage inducible transcript 3 (*Ddit3*). (d) relative mRNA expression levels of inflammation-related genes in eWAT: tumor necrosis factor alpha (*Tnfα*), chemokine (C-C motif) ligand 2 (*Ccl2*), interleukin 6 (*iIl-6*), Lipocalin 2 (*Lcn2*), Adiponectin (*Adipoq*) and leptin (*Lep)*. Data are mean ± SEM. ^$^*p <0.05*, ^#^*p <0.01*, ^¤^*p <0.001 versus* the Chow diet group and *p <0.05, **p <0.01, ***p <0.001 for the HF-Pr2 v*ersus* the HF group.

Compared to the Chow diet, 12 weeks on the HF diet induced a concerted increase in the mRNA levels of a set of genes involved in the handling and storage of lipids in the liver and adipose tissue (Figure 2a,b), such as *LipC* (encoding hepatic lipase), *Lpl* (lipoprotein lipase) and *Ldl-r* (LDL receptor), *Cd36* (encoding fatty acid transporter) and *Dgat2* (diacylglycerol O-acyltransferase 2, catalyzing the final reaction in the synthesis of triglycerides). These findings are in line with the lipid accumulation observed in hepatic and adipose tissues. Supplementation with the probiotic mix reduced the expression of *LipC* in the liver (−30%) and of *Ldl-*r in eWAT (−60%) compared to the HF diet-fed group (Figure 2a,b). The relative abundance of mRNA for *Cd36* fatty acid transporter also decreased (by almost 50%) upon probiotics in the liver. With regard to genes related to hepatic *de novo* lipogenesis, *Srebp-1c*, *Fasn*, and *Dgat2* expression was significantly reduced in the HF-Pr2 group compared with the HF group. Furthermore, in the eWAT, supplementation with the probiotic mix was associated with a significant increase in the abundance of *Hsl* (hormone-sensitive lipase) and *Atgl* (adipose triglyceride lipase) mRNA, the genes encoding the main lipolysis enzymes (Figure 2a,b).

To further explore possible mechanisms explaining the probiotic mix-induced reduction of weight gain and adipose tissue accumulation, the expression of genes related to brown fat activation and white adipose tissue browning was evaluated in fat depots, as well as the expression of genes related to fatty acid oxidation in the liver. These different pathways were not affected by probiotic mix intake, although the HF diet was associated with an increase in the expression of the uncoupling protein 1 (*Ucp1*) gene in brown fat (Supplementary Figure S2).

Accumulation of lipids in the liver is classically associated with endoplasmic reticulum (ER) stress and oxidative stress. Accordingly, we observed that the HF diet induced a significant increase in the expression of the anti-oxidant *Gpx1* and *Cat* genes and the ER stress markers *Hspa5* (encoding the chaperone Bip) and *Ddit3* (encoding the C/EBP homologous protein Chop) (Figure 2c). Supplementation with the probiotic mix completely prevented these HF-induced effects. Furthermore, the expression of a set of additional anti-oxidant genes (*Gpx4*, *Prdx3*, *Sod2*, and *Gsta4*) were significantly reduced in the HF-Pr2 group compared with the HF group (Figure 2c), clearly indicating a protective effect of the probiotic mix on oxidative and ER stresses in the liver of mice fed a HF diet.

The expansion of adipose tissue upon a hypercaloric diet is generally associated with an increase in inflammatory markers, reflecting modifications in the population of macrophages and lymphocytes infiltrating the tissue.^5^ In agreement, we observed an increase in the expression of TNFα and the chemokine MCP-1 (coded by *Ccl2* gene) in eWAT (Figure 2d). The pro-inflammatory adipokine leptin (encoded by *Lep* gene) was also markedly overexpressed in HF diet-fed mice (Figure 2d). Importantly, supplementation with the probiotic mix strongly prevented HF-induced expression of inflammatory markers in eWAT (Figure 2d), consistent with its protective effect against fat accumulation.

### Probiotic mix supplementation was associated with a reduction in postprandial plasma triglycerides and lower expression of genes involved in fatty acid uptake in the small intestine

**Figure 3.**
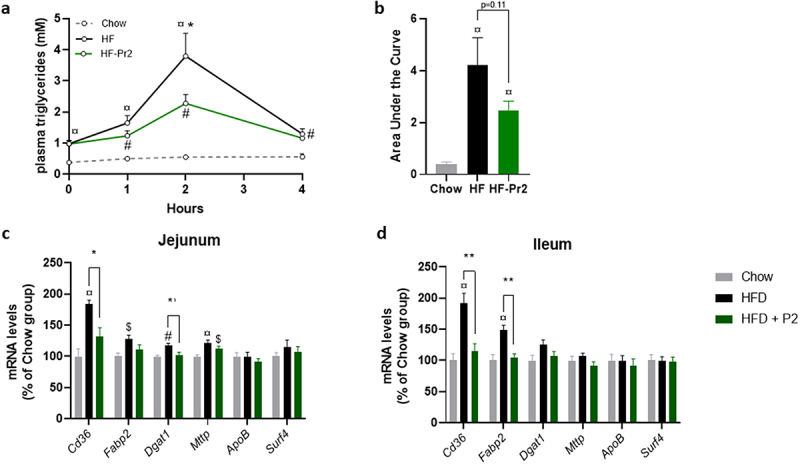
Effects of supplementation with the probiotic mix on intestinal lipid uptake. (a) plasma triglyceride concentrations throughout the oral lipid tolerance test (OLTT) (*n* = 9–11). (b) area under the curve (AUC) of plasma TGs during OLTT. Relative mRNA expression levels of lipid absorption-related genes in the jejunum (c) and ileum (d) *Cd36*, fatty acid binding protein 2 (*Fabp2*), diacylglycerol O-acyltransferase 1 (*Dgat1*), Microsomal triglyceride transfer protein (*Mttp)*, apolipoprotein B (*ApoB)* and Surfeit4 (*Surf4)* (*n* = 7–10). Data are mean ± SEM. ^$^*p <0.05*, ^#^*p <0.01*, ^¤^*p <0.001 versus* the Chow diet group and *p <0.05, **p <0.01 for the HF-Pr2 *versus* the HF group.

The fact that the probiotic mix prevented weight gain and adipose tissue accumulation without significantly modifying food intake and lipid oxidation pathways the liver in brown adipose tissue (BAT) could suggest an impact of the probiotic mix on lipid absorption. This hypothesis was tested using an oral lipid tolerance test (OLTT) performed after 10 weeks of supplementation. During the 4 h following gavage of mice with oil, plasma concentrations of TG were significantly higher in the HF group than in the Chow diet fed group, peaking 2 h after gavage and returning almost to basal values after 4 h (Figure 3a). Interestingly, supplementation with the probiotic mix was associated with a significantly lower TG peak at 2 h (*p = 0.033* between the HF-Pr2 and HF groups, Figure 3a). The areas under the curves (AUCs) of postprandial plasma TG were significantly higher in the HF and HF-Pr2 groups than in the Chow diet fed group, and there was a trend (*p = 0.11*) toward a reduction in the group supplemented with the probiotic mix compared with HF diet-fed mice (Figure 3b). These results might suggest a reduction of lipid absorption in the presence of the probiotic mix. However, the postprandial excursion of TG during OLTT results from both intestinal absorption of lipids and their clearance from plasma. The lack of an increase in circulating TG after the oil bolus in Chow diet mice suggests a highly efficient rate of TG clearance in this group and thus potentially impaired clearance in the HF diet groups. To better clarify this point, we evaluated the expression of key genes involved in fatty acid uptake and chylomicron production in the jejunum and the ileum, the two main regions of the small intestine involved in the absorption of dietary lipids. The expression of *Cd36* (allowing transport of fatty acids from the lumen to the enterocytes) and *Fabp2* (encoding an intracellular transporter of fatty acids) was increased in both the jejunum and ileum of HF diet-fed mice compared to Chow diet-fed mice. Supplementation with the probiotic mix prevented the overexpression of *Cd36* in both tissues (Figure 3c,d). However, other key genes of lipid absorption linked to intracellular TG synthesis (*Dgat1*) and chylomicron production by enterocytes (*Mttp, ApoB*, and *Surf4*) were globally not affected (Figure 3c,d).

### Probiotic mix supplementation did not affect intestinal permeability and was associated with some modifications of gut microbiota composition

Since increased intestinal permeability has been associated with inflammation and metabolic disturbances in rodent models fed high-fat diets,^12^ we evaluated this parameter *in vivo* using the lactulose-mannitol test. While HF diet-fed mice showed a clear alteration in the integrity of the intestinal barrier, as illustrated by a large increase in the recovery of lactulose in urine, supplementation with the probiotic mix did not correct the HF diet-induced alteration of intestinal permeability (Supplementary Figure S3). In addition, mRNA expressions of *Muc2* and *Tjp1* (encoding the tight junction protein ZO1) in the ileum were not affected by the probiotic mix consumption (Supplementary Figure S3).

**Figure 4.**
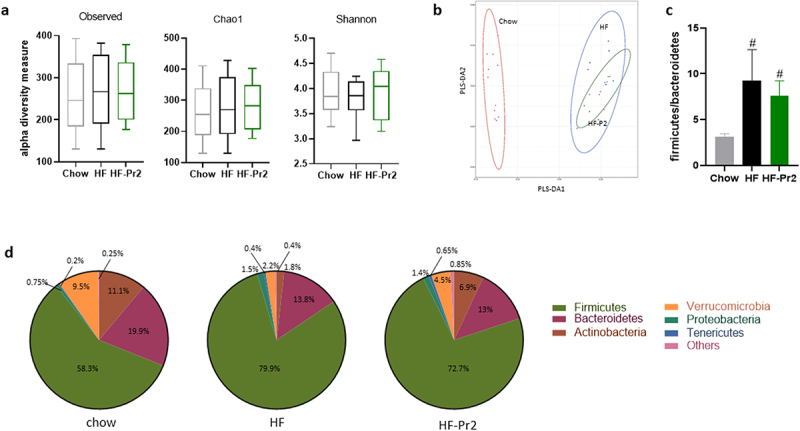
Impacts of supplementation with the probiotic mix on gut microbiota composition. (a) alpha-diversity based on observed OTUs, Chao1 index and Shannon indexes. (b) multidimensional scaling of Bray-Curtis dissimilarity (MDS). (c) Firmicutes/Bacteroidetes ratio. (d) pie charts showing relative abundance of the most abundant *phyla*. Data are mean ± SEM. ^#^*p <0.01 versus* the Chow diet group.

Gut microbiota composition was analyzed by sequencing of the V3-V4 region of 16S rRNA in feces collected at the end of the protocol. A total of 766 different amplicon sequence variants (ASVs) were identified. Alpha diversity estimated using different indexes did not differ between groups (Figure 4a). However, a classical multidimensional scaling (MDS) using Bray-Curtis dissimilarity indicated that mice fed the HF diet had a different microbiota structure and composition than those fed the Chow diet, but this was not corrected by probiotic mix intake (Figure 4b). After taxonomic assignment, comparisons between groups at the *phylum* level showed that mice fed the HF diet had increased abundance of Firmicutes (now called Bacillota) and reduced levels of Bacteroidetes (Bacteroidota), Actinobacteria (Actinomycetota), and Verrucomicrobia (Figure 4d). The Firmicutes/Bacteroidetes (Bacillota/Bacteroidota) ratio was significantly increased by the HF diet and not corrected by the probiotic mix (Figure 4c). The only significant difference observed at the phylum level between HF diet-fed mice and probiotic mix-supplemented mice was an increase in the abundance of Actinobacteria (Actinomycetota) in the HF-Pr2 group (6.9% vs 1.8%, *p* = 0.0096, Figure 4d).

At the *genus* level, the HF diet induced several changes in the composition of gut microbiota when compared with the Chow diet (Supplementary Table S1). The relative abundance of 12 *genera* were significantly modified by the HF-diet: five were decreased (*Akkermansia*, *Bifidobacterium*, *Coprococcu*s, *Ruminococcus*, and *Turicibacter)* and seven increased (*Adlercreutzia*, *Allobaculum*, *Bilophila*, *Gemmiger*, *Mucispirillum*, *Oscillospira*, and *Streptococcus)*. However, the relative abundance of only two *genera* was significantly modified by the probiotic mix when compared to the HF diet-fed group: a decrease in *Adlercreutzia* (−2.8-fold) and an increase in *Bifidobacterium* (7.5-fold) (Supplementary Table S1).

Among the 766 ASVs, 301 showed significant differences between groups according to Kruskal–Wallis tests with multiple corrections. When comparisons between groups were performed using pairwise Wilcoxon tests, 269 ASVs had different relative abundances between the Chow diet and HF-diet groups and 277 between the Chow diet and HF-Pr2 groups. Only 17 ASVs displayed significantly different levels between the HF diet and the HF diet plus probiotic mix groups, 7 being increased and 10 being decreased in response to supplementation with the probiotic mix (Table 1). We then manually verified the assignments of these 17 ASVs (using BLAST-NCBI), which allowed us to better identify the bacteria affected by supplementation with the probiotic mix. We found that the largest increases in relative abundance were for *Bifidobacterium animalis* subsp. *lactis* (ASV_79) and *Lactobacillus gasseri* (ASV_673) (Table 1), in agreement with consumption of LA804 and LA806, the two strains of the probiotic mix. Among the other up-regulated ASVs, we found ASV_418 (*Olsenella*) and ASV_379 (*Lawsonibacter*), which were specifically induced in the presence of the probiotic mix and not modified by the HF diet itself (Supplementary Figure S4). The three other ASVs with a significant increase in relative abundance ASV_449 (*Christensenella*), ASV_87 (*Roseburia*) and ASV_233 (*Flintibacter butyricus*) were more than twofold higher in the HF-Pr2 group than in the HF-diet group (Table 1), but they were also increased by the HF diet *per se* (Supplementary Figure S4).

**Table 1.** Description of the 17 ASVs with significant change in relative abundance in the feces of the probiotic-treated mice compared to HF fed mice.

ASV	Phylum	Class	Order	Family	Genus	Species	K-W corrected p-value	Pairwise Wilcoxon p-value	Fold change (HF-Pr2 *vs* HF)
79	*Actinobacteria*	*Actinobacteria*	*Bifidobacteriales*	*Bifidobacteriaceae*	*Bifidobacterium*	*animalis subsp. lactis (100%)*	0.00125	0.00007	42469
673	*Firmicutes*	*Bacilli*	*Lactobacillales*	*Lactobacillaceae*	*Lactobacillus*	*gasseri (100%)*	0.00111	0.00007	1313
418	*Actinobacteria*	*Coriobacteriia*	*Coriobacteriales*	*Atopobiaceae*	*Olsenella*	N.A.	0.00228	0.00048	8.6
379	*Firmicutes*	*Clostridia*	*Eubacteriales*	*Oscillospiraceae*	*Lawsonibacter*	N.A.	0.01317	0.00658	7.5
449	*Firmicutes*	*Clostridia*	*Eubacteriales*	*Christensenellaceae*	*Christensenella*	N.A.	0.00193	0.01282	2.8
87	*Firmicutes*	*Clostridia*	*Eubacteriales*	*Lachnospiraceae*	*Roseburia*	N.A.	0.00760	0.02331	2.5
233	*Firmicutes*	*Clostridia*	*Eubacteriales*	*Eubacteriales incertae sedis*	*Flintibacter*	*butyricus (100%)*	0.00186	0.03078	2.1
137	*Actinobacteria*	*Actinomycetes*	*Bifidobacteriales*	*Bifidobacteriaceae*	*Bifidobacterium*	*pseudolongum (100%)*	0.00111	0.02665	−1.9
276	*Actinobacteria*	*Coriobacteriia*	*Eggerthellales*	*Eggerthellaceae*	*Adlercreutzia*	*muris (100%)*	0.00111	0.00032	−2.6
484	*Firmicutes*	*Clostridia*	*Eubacteriales*	*Lachnospiraceae*	*Anaerostipes*	N.A.	0.04651	0.03997	−5.9
736	*Firmicutes*	*Clostridia*	*Eubacteriales*	*Lachnospiraceae*	*Enterocloster (or Clostridium)*	N.A.	0.00185	0.03428	not detectable in HF-Pr2
301	*Bacteroidetes*	*Bacteroidia*	*Bacteroidales*	*Muribaculaceae*	*Muribaculum*	N.A.	0.00111	0.04342	not detectable in HF-Pr2
17	*Firmicutes*	*Clostridia*	*Eubacteriales*	*Lachnospiraceae*	*Herbinix (or Anaerobium)*	N.A.	0.00160	0.03428	not detectable in HF-Pr2
554	*Firmicutes*	*Clostridia*	*Eubacteriales*	*Lachnospiraceae*	*Dorea*	*formicigenerans (97%)*	0.01359	0.00411	not detectable in HF-Pr2
152	*Firmicutes*	*Clostridia*	*Eubacteriales*	*Oscillospiraceae*	*Acetivibrio*	*thermocellum (89%)*	0.01241	0.00617	not detectable in HF-Pr2
438	*Firmicutes*	*Clostridia*	*Eubacteriales*	*Lachnospiraceae*	*Fusimonas*	*intestini (97%)*	0.03763	0.02331	not detectable in HF-Pr2
235	*Firmicutes*	*Clostridia*	*Eubacteriales*	*Lachnospiraceae*	*Simiaoa*	N.A.	0.03951	0.01810	not detectable in HF-Pr2

Of the bacteria whose relative abundance was reduced by supplementation with the probiotic mix, six belong to the *Lachnospiraceae* family, including bacteria belonging to *genera* previously associated with obesity or diabetic phenotypes in rodents and humans,^21–24^ such as *Dorea* (ASV_554), *Fusimonas* (ASV_438), or *Enterocloster* (ASV_736) (Table 1). Interestingly, these bacteria were not detectable in the feces of mice supplemented with the probiotic mix (Table 1). Furthermore, four ASVs appeared to be of great interest with respect to the benefit of supplementation with the probiotic mix, since their relative abundance was oppositely regulated by the probiotic mix compared to the HF diet alone. ASV_235 (*Lachnospiraceae Simiaoa*), ASV_152 (*Acetivibrio*), ASV_554 (*Dorea*), and ASV_276 (*Adlercreutzia muris*) were induced by the HF diet, and supplementation with the probiotic mix was able to prevent these inductions (Supplementary Figure S5). The other ASVs whose relative abundance in feces was lower in probiotic mix-supplemented mice than in HF diet-fed mice corresponded to bacteria already strongly down-regulated in HF diet-fed mice compared to Chow diet fed mice, leading to undetectable levels in mice supplemented with the probiotic mix, as seen for ASV_137 (*Bifidobacterium pseudolongum)*, ASV_301 (*Muribaculum*), ASV_438 (*Fusimona*s) or ASV-17 (*Herbinix* or *Anaerobium*) (Supplementary Figure S5). For this subgroup of bacteria, it is likely that the observed reduction in relative abundance did not contribute to the beneficial action of probiotic supplementation since the changes appeared mainly due to the HF diet.

To gain more insight on how modifications of the composition of the microbiota in the presence of the probiotic mix could have had an impact on host metabolism, we first focused on changes in the concentration of SCFAs in feces because several modified bacteria are potential SCFA producers. However, quantification of the main SCFAs did not reveal any significant effects of probiotic intake. In fact, while the HF diet was associated with a coordinated reduction of the four measurable SCFAs, the intake of the probiotic mix did not modify their concentrations in feces (Supplementary Figure S6).

### Probiotic mix supplementation downregulated the FXR-Fgf15 pathway in the ileum

**Figure 5.**
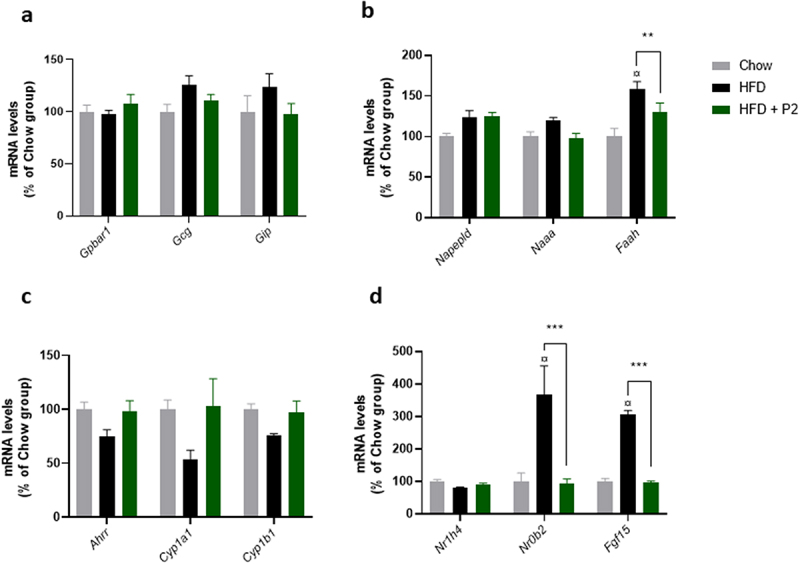
Effects of supplementation with the probiotic mix on regulatory pathways in the ileum. (a) incretin pathway. Relative mRNA expression levels of G protein-coupled bile acid receptor 1 (*Gpbar1*), glucagon (*Gcg*) and gastric inhibitory polypeptide (*Gip*) in the ileum. (b) endocannabinoid pathway. Relative mRNA expression levels of N-acyl phosphatidylethanolamine phospholipase D (*Napepld*), N-acylethanolamine acid amidase (*Naaa*) and fatty acid amide hydrolase (*Faah*) in the ileum. (c) Ahr pathway. Relative mRNA expression levels of Aryl-hydrocarbon receptor repressor (*Ahrr*), cytochrome P450 family 1 subfamily a polypeptide 1 (*Cyp1a1*) and cytochrome P450 family 1 subfamily b polypeptide 1 (*Cyp1b1*) in the ileum. (d) FXR-Fgf15 pathway. Relative mRNA expression levels of nuclear receptor subfamily 1 group H member 4 (*Nr1h4*), nuclear receptor subfamily 0 group B member 2 (*Nr0b2*) and fibroblast growth factor 15 (*Fgf15*) in the ileum. Data are mean ± SEM (*n* = 7–10). ^¤^*p <0.001 versus* the Chow diet group and **p <0.01, ***p <0.001 for the HF-Pr2 *versus* the HF group.

To further investigate whether other intestinal processes could be affected by supplementation with the probiotic mix, we assessed in the ileum, the expression of different sets of genes involved in important metabolic or hormonal pathways previously associated in the literature with beneficial responses to probiotics. A number of lactic bacteria have been shown to stimulate the production of the incretin hormones GIP (glucose-dependent insulinotropic polypeptide) and GLP1 (glucagon-like peptide 1, encoded by the *Gcg* gene), and of TRG5 receptor (encoded by *Gpbar1*) in intestinal L cells.^25–28^ The expression of these genes was not affected in the ileum (Figure 5a). In the jejunum, *Gcg* and *Gip* mRNA levels were induced by the HF diet. The probiotic mix only reduced the expression level of *Gip* (Supplementary Figure S7).

We then measured the expression of genes involved in the synthesis (*Napepld*) and the degradation (*Naaa* and *Faah*) of endocannabinoids and N-acetylamines, important biomolecules involved in several biological processes, including the control of food intake and inflammation.^29^ The expression of *Faah* was induced by the HF diet, this overexpression being prevented by the probiotic mix, whereas the other tested genes were not significantly affected (Figure 5b). Because *Faah* gene is coding for the fatty acid amide hydrolase that catalyzes the degradation of both anandamide (AEA) and other N-acetylamines, and to a lesser extend 2-arachidonoylglycerol (2-AG), we measured their concentrations in the small intestine. The concentrations of AEA and 2-AG were not significantly different between groups (Supplementary Figure S8). Regarding N-acetylamines, stearoylethanolamide (SEA) concentration was increased by the HF diet but not affected by the probiotic mix (Supplementary Figure S8).

Recently, some *Lactobacillus* strains have been reported to improve metabolic homeostasis in HF diet-fed mice through the modulation of indole derivatives in the gut, leading to stimulation of the intestinal AhR pathway.^26^ We measured the expression of three classical target genes of the nuclear receptor AhR in the ileum (*Ahrr*, *Cyp1a1*, and *Cyp1b1*). Despite a trend toward a concerted reduction of their expression in the HF-diet group compared to the Chow diet fed group and an up-regulation with the probiotic mix, the differences observed between groups were not statistically significant (Figure 5c).

Finally, we investigated the regulation of the FXR-FGF15 pathway in the ileum. The intestinal bile acid-activated nuclear receptor FXR (farnesoid X receptor, coded by the *Nr1h4* gene) has emerged as a major player in metabolic regulation.^30^ FXR transcriptional activity was stimulated during the HF diet, as evidenced by a strong induction of its two major target genes *Fgf15* (coding for the fibroblast growth factor 15) and *Nrob2* (coding for the small heterodimer partner SHP) (Figure 5d). Supplementation with the probiotic mix completely prevented the overexpression of these two genes, thus supporting an inhibition of FXR activity in the ileum. The expression of *Nr1h4* (coding FXR) itself was not affected by the diets (Figure 5d).

### Probiotic mix supplementation modified bile acid profile favoring FXR antagonists in the caecum

**Figure 6.**
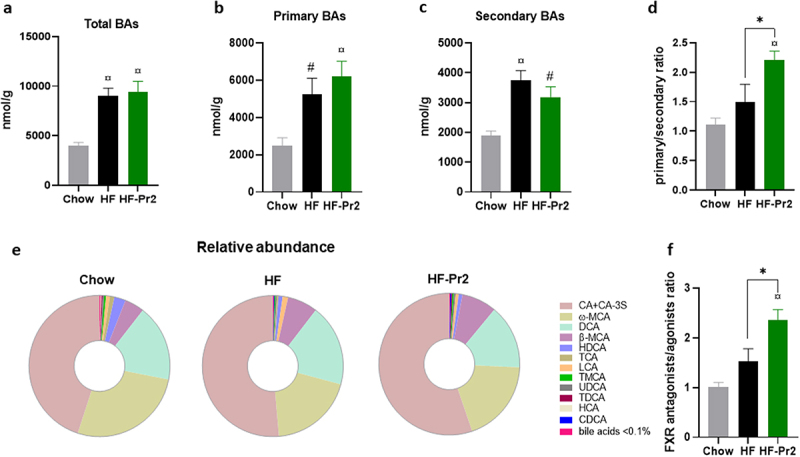
Effects of the probiotic mix on bile acid (BA) profile in the caecum. (a) concentration of total BAs in the caecum. (b) amount of primary BAs (CA, CA-3S, CDCA, TCDCA, TCA, HCA, β-MCA and TMCA). (c) amount of secondary BAs (DCA, TDCA, LCA, ω-MCA, HDCA and THDCA). (d) primary and secondary BA ratio. (e) BA profile in the caecum. (f) FXR antagonists/FXR agonists ratio. Data are mean ± SEM (*n* = 6–10). ^#^*p <0.01*, ^¤^*p <0.001 versus* the Chow diet group and *p <0.05 for the HF-Pr2 *versus* the HF group.

Intestinal FXR activity is directly under the control of BA molecules present in the gut lumen. We therefore determined, by targeted LC-MS/MS, the concentrations of the main BAs in the caecum. The total BA concentration was significantly increased in HF diet-fed mice compared to Chow diet fed mice (Figure 6a). Supplementation with the probiotic mix did not affect the total amount but tended to increase the concentration of primary (Figure 6b) and to reduce the amount of secondary (Figure 6c) BAs, leading to a significant increase in the primary to secondary BA ratio (Figure 6d). The BA pool composition was moderately modified by the HF diet, with an increase in cholic acid (CA and its related compounds, mainly CA 3-sulfate), deoxycholic acid (DCA), β-muricholic acid (βMCA), and lithocholic acid (LCA) levels in the caecum of HF diet-fed mice (Figure 6e and Supplementary Figure S9). No major differences in the BA profile were observed between HF diet-fed and HF-Pr2 mice, except for LCA levels that were significantly reduced with the probiotic mix (Supplementary Figure S9). Among the different BAs, several can stimulate the transcriptional activity of FXR (such as CA, DCA, CDCA, LCA, and ωMCA), while others are well-described antagonists of intestinal FXR (especially β-MCA, TMCA, UDCA, and TUDCA). It is then important to note that the ratio of FXR antagonist/agonist BAs was significantly higher in HF-Pr2 mice than in HF diet-fed mice (Figure 6f), in line with the observed reduction of FXR activity in the ileum of mice supplemented with the probiotic mix.

Modulations of the BA profile in the gut could result from changes in the rate of their production and secretion by the liver and/or from enzymatic modifications by bacterial enzymes in the intestinal lumen. The mRNA levels of key genes of BA synthesis, modification, and export from hepatocytes were not different in the liver of HF diet-fed mice with or without probiotic supplementation (Supplementary Figure S10). The hepatic expression of *Cyp7a1*, coding the rate-limiting enzyme of the classical pathway, was significantly increased in the two groups fed with the HF diet (Supplementary Figure S10), in agreement with the higher levels of CA and total BAs observed in the gut of the HF diet-fed mice.

### Inhibition of intestinal FXR pathway was associated with improvement of host phenotype markers during supplementation with the probiotic mix

**Figure 7.**
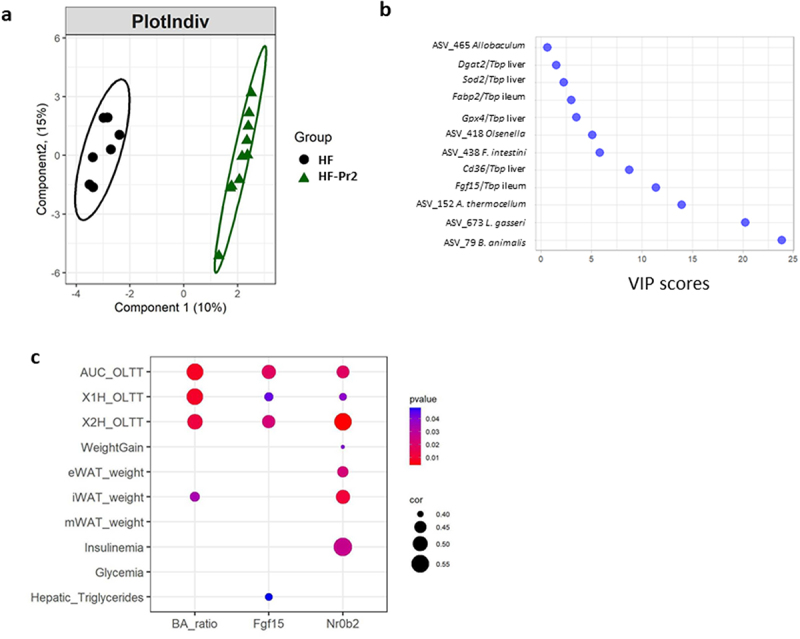
A multivariate approach highlights significant separation between HF and HF-Pr2 groups and the potential role played by the inhibition of intestinal FXR pathway in the beneficial effects of the probiotic mix. (a) partial least squares-discriminant analysis (PLS-DA) plot of individual mice from HF and HF-Pr2 groups and (b) Variable Importance in the project (VIP) representation of the most discriminant features identified by PLS-DA. (c) correlation plot of indexes of intestinal FXR inhibition (Ba_ratio = ratio of FXR antagonist/agonist bile acid species), Fgf15 (*Fgf15*/*Tbp* mRNA ratio measured in the ileum) and Nr0b2 (*Nr0b2*/*Tbp* mRNA ratio measured in the ileum) and MetS related parameters (X1H_OLTT and X2H_OLTT correspond to the plasma TG values measured during the OLTT 1 and 2 hours after administration of the oil bolus). Spearman correlation coefficients are indicated by the size of the dots, p-values are indicated by the color.

When the whole dataset recorded and/or generated in the present study was taken into account (*n* = 1474 variables), a Partial Least-Squares Discriminant Analysis (PLS-DA) clearly separated the HF and the HF-Pr2 groups (Figure 7a). The Variable Importance in Projection (VIP) score identified the changes in the abundance of the two probiotic strains (ASV_79 and ASV_673), of ASV_152 (*Acetivibrio*), and that of *Fgf15* expression in the ileum, as the four most discriminant parameters between HF and HF-Pr2 mice (Figure 7b). This analysis thus highlighted the reduction of *Fgf15* expression, and therefore the inhibition of FXR activity in the ileum, as a major contributor to the differences between the two groups. To verify whether intestinal inhibition of the FXR pathway could be associated with improved anthropometric and metabolic parameters, we looked for correlations between indicators of intestinal FXR activity (i.e. the FXR antagonist to agonists bile acid ratio and the gene expression of *Fgf15* and of *Nr0b2* in the ileum) and the different variables related to obesity and MetS (weight gain, adipose tissue weights, liver triglycerides, results of OLTT, insulinemia, and glycemia) in the three groups of mice. The intestinal FXR activity indicators were significantly correlated with OLTT data (AUC and peaks of TG after 1 and 2 h) (Figure 7c). The expression level of *Nr0b2* was correlated with eWAT and iWAT weights. When Benjamini–Hochberg (BH) multiple correction was applied, only the correlation with the AUC of OLLT remained significant (Supplementary Figure S11). We also identified significant correlations between *Fgf15* and *Nr0b2* gene expression in the ileum and the expression of *Cd36* and *Fabp2*, key genes of fatty acid uptake in ileal and jejunal enterocytes (Supplementary Figure S12).

## Discussion

The use of probiotics to help ameliorate obesity and associated metabolic disorders is now considered a valid and promising strategy.^14,15,18^ *Lactobacillus* and *Bifidobacterium* strains are commonly used for this purpose, and a number of studies have clearly evidenced the beneficial effects of such strains on obesity^14^ and, for some of them, on the characteristics of Mets.^15–17^ However, the multiple possible mechanisms of action involved in the improvement of metabolic health are complex and generally remain to be clarified.^31^ Furthermore, the beneficial effects often appear to be related to the individual properties of specific strains rather than to a general probiotic effect.^32^ The validation and characterization of the mechanisms of action of effective strains are therefore of the utmost importance. Using a multi-criteria *in vitro* screening strategy followed by *in vivo* tests in pre-clinical models, Alard and colleagues identified a high-potential mixture of *B. animalis subsp. lacti*s LA804 and *L. gasseri* LA806 strains, having a significant impact on obesity in HF diet-fed mice^20^. In the present study, we further characterized the potential of the combination of these two strains to prevent MetS, obesity onset, and lipid metabolism dysfunction in mice. We identify the contribution of an inhibition of the intestinal FXR pathway as a possible mechanism to explain the beneficial effects of these probiotics.

We first confirmed that the supplementation with the probiotic mix limited body weight gain and adipose tissue accumulation, without modifying food intake, in HF diet-fed mice. These effects were accompanied by protection against inflammation in the adipose tissue and against lipid accumulation, oxidative stress, and ER stress in the liver. These beneficial effects on various hallmarks of MetS and obesity were not associated with a reorientation of the fate of excessive lipid intake during the HF diet toward catabolic pathways such as brown fat stimulation or hepatic mitochondrial and peroxisomal oxidations. The browning of WAT was recently proposed to contribute to the effects of *L. amylovorus* KU4 (*Amylolactobacillus amylophilus*),^33^ but here we did not find any evidence of modification of browning markers after intake of the probiotic mix. To better characterize the effects on lipid metabolism, we evaluated the impact of the probiotic mix on postprandial lipemia *in vivo* using an oral lipid tolerance test. We found that the postprandial plasma TG peak was lowered during OLTT in mice treated with the probiotic mix compared to the HF diet-fed mice. At the molecular level in the jejunum and ileum, the probiotic mix protected against HF-induced rise in mRNA levels of *Cd36 and Fabp2*, two transporters involved in fatty acid uptake by enterocytes. These results suggest that the pathways involved in intestinal lipid uptake are globally modulated by the consumption of the probiotic mixture. Nevertheless, since OLTT results are the balance between lipid absorption and TG clearance from the plasma, the impacts of the probiotic mix on these mechanisms, and to which extent they contribute to lowering the accumulation of fat in tissues and improving metabolic parameters, remains to be elucidated.

Various mechanisms have been proposed to explain the beneficial effects of probiotics on metabolic health, including improvement of the gut barrier function.^34^ Increased intestinal permeability is considered a major defect in obesity and MetS, participating in endotoxin translocation from gut lumen to circulation and induction of a pro-inflammatory state in the adipose tissue, liver, and other peripheral organs.^35^ Some probiotics have been reported to restore intestinal permeability in *in vitro* models, animal models, and, in some cases, in humans.^34,36^ Here, in agreement with numerous studies in rodents,^37^ we found that the HF diet was associated with a significant increase in gut permeability assessed *in vivo* by the lactulose-mannitol test. However, the probiotic mix did not protect or improve the HF diet-induced leaky gut. This important result demonstrated that, under our experimental conditions, improvement of intestinal permeability was not required for the beneficial action of the tested probiotic mix on obesity, metabolic health, and lipid metabolism.

Another classical mechanism proposed to explain the beneficial actions of probiotics is the production of SCFAs,^38^ either directly by the probiotic strains able to synthesize them or by modulation of the gut microbiota composition favoring SCFA-producing bacteria. Although *Lactobacillus* and *Bifidobacteria* are known producers of SCFAs and their consumption in the present study was accompanied by an increase in the relative abundance of other species able to produce SCFAs, including *Olsenella*,^39^ *Lawsonibacter*,^40^ *Christensenella* ,^41^ *Roseburia*,^42^ and *Flintibacter butyricus*,^43^ we were surprised to find that the supplementation with the probiotic mix was not associated with a change in the levels of the main SCFAs (acetate, propionate, butyrate, and valerate) in fecal samples. Indeed, the HF diet induced, as expected, a strong reduction in the levels of these four molecules, but this effect was not prevented or restored by the probiotic mix. However, we cannot exclude that changes in SCFAs have occurred in other compartments such as in plasma or caecum. It has recently been demonstrated that circulating rather than fecal SCFAs are more directly linked to metabolic health in a cross-sectional study in humans.^44^ One of the described beneficial effects of SCFAs on metabolic dysfunctions is the stimulation of the production of incretin hormone GLP1 by intestinal endocrine cells.^45^ Consistent with the absence of SCFA modifications in this study, no changes in the expression of *Gcg* gene (coding for GLP1) were found in the ileum and jejunum of supplemented mice.

Improved metabolic health in HF-fed mice was reported after administration of *Limosilactobacillus reuteri*, which is able to produce indoles and their derivatives that can activate the aryl hydrocarbon receptor (AhR).^26^ These AhR agonists are microbiota metabolites derived from tryptophan and are strongly reduced in the gut lumen and feces in obesity and MetS.^26^ Activation of AhR by indoles in the small intestine is associated with the restoration of gut barrier defects and the production of GLP1.^26^ Although these two parameters were not modified in the study, we assessed the possible involvement of the AhR pathway by evaluating the expression of classical target genes of Ahr in the ileum (*Ahrr*, *Cyp1a1*, and *Cyp1b1)*. The lack of difference between groups further supported that the AhR pathway did not play a major role in this study.

Another potential mechanism involved in the positive effects of the probiotic mix may be the modulation of the endocannabinoid system in the gut. An interplay between metabolic health, intestinal barrier integrity, and endocannabinoid system balance has already been demonstrated,^13^ and some studies have reported effects of probiotics on endocannabinoid concentration in the ileum, such as an increase in 2-AG, after administration of *Akkermansia muciniphila* in HF-diet fed mice, which was associated with improved metabolic dysfunctions.^46^ While we observed a modification of *Faah* gene expression, the enzyme involved in the degradation of endocannabinoids, in the ileum of the supplemented mice, we did not find any modifications in the concentration of anandamide and the main N-acetylamines in small intestinal tissues.

FXR plays a pivotal role in the metabolism of BA, lipids, and glucose, but also in inflammation and intestinal barrier function.^30,47^ In the ileum, FXR controls the expression of Fgf15 (fibroblast growth factor 15 in rodent and FGF19 in humans), an important regulator of glucose and lipid homeostasis^48^ in addition to its role in the control of BA synthesis in the liver.^49^ BAs are natural ligands of FXR and can be either activators or inhibitors, with CDCA (chenodeoxycholic acid) and DCA (deoxycholic acid) being the more potent agonists, whereas UDCA (ursodeoxycholic acid) and β-MCA (muricholic acid) are antagonists of FXR. Supplementation of HF diet-fed mice with the probiotic mix did not affect the total amount of BAs but led to a significant increase in the primary to secondary BA ratio in the caecum and a reduction in LCA abundance. Furthermore, we found a significant increase in the FXR antagonists/agonists ratio, in line with a marked inhibition of FXR activity in the ileum of the supplemented mice, as evidenced by a drastic reduction in the expression of the FXR target genes *Fgf15* and *Nr0b2*. Interestingly, it was recently reported that inhibition of intestinal FXR activity by caffeic acid phenethyl ester specifically in the gut exerts potent anti-diabetic effects.^50^ Similarly, increased levels of intestinal *T*-β-MCA (the tauro-conjugated β-muricholic acid) which is a strong antagonist of FXR has been shown to improve HFD-induced obesity, hepatic steatosis, and glucose intolerance *via* the inhibition of intestinal FXR activity.^51^ In addition, the interplay between BA pool composition, intestinal FXR activity, and lipid absorption has recently been highlighted.^52^ Altogether, these data indicate that an inhibition of intestinal FXR signaling could be involved in the mechanisms of action of the probiotic mix tested in this study.

Using a PLS-DA with all the parameters and data measured in the study (1474 variables), we found that the decrease in the expression of *Fgf15* in the ileum, reflecting the inhibition of intestinal FXR activity, was among the most discriminant variables between the HF and the HF-Pr2 groups. Furthermore, the changes in the expression of *Fgf15* and *Nrob2* and of the FXR antagonist/agonist bile acid ratio were correlated with the evolution of the postprandial lipemia assessed by OLTT and, to a lower extent, to the size of adipose tissue depots. *Fgf15* and *Nr0b2* expressions were also correlated with small intestinal expression of *Cd36* and *Fabp2*, key actors of fatty acid uptake by enterocytes. Altogether, these data are thus indicative of the contribution of the inhibition of intestinal FXR signaling in the mechanisms of action of the tested probiotic mix in preventing obesity and metabolic deteriorations upon HF diet, potentially through a limitation of intestinal fatty acid uptake.

Recent works have demonstrated the potential of the inhibition of FXR activity specifically in the small intestine to counteract obesity and to improve metabolic disturbances associated with diabetes and nonalcoholic fatty diseases.^47,51,53^ This original concept derived from the observation that intestine-specific *Fxr* gene invalidation in the mouse protects against diet-induced obesity.^51^ Contradictory results have been reported in this genetically modified model showing, for example, exacerbation of alcoholic fatty liver disease in intestinal FXR deficient mice.^54^ However, the use of inhibitors or antagonists acting selectively on intestinal FXR, such as caffeic acid phenethyl ester, *T*-β-MCA, or glycine-β-MCA, clearly supports a beneficial role of the inhibition of FXR.^50,51,55^ Clinical trials with UDCA, a strong FXR antagonist, also showed an increase in insulin sensitivity in patients with nonalcoholic steatohepatitis or obesity.^47^ Furthermore, it was demonstrated that the beneficial effect of metformin in type 2 diabetic patients is associated with a change in the intestinal BA profile, favoring FXR antagonists (especially GUDCA) and the inhibition of FXR signaling.^56^ We recently found that a similar mechanism may occur in mice treated with metformin.^57^

Little is known regarding the possibility that probiotics could specifically target intestinal FXR signaling to improve metabolic health. It was shown that the probiotic mix VSL#3 was able to down-regulate the FXR-Fgf15 pathway via modulation of BA metabolism, enhancing in turn BA deconjugation and their fecal excretion.^58^ VSL#3 is currently sold as a mixture of eight different strains of *Lactobacillus*, *Bifidobacterium*, and *Streptococcus thermophilus* BT01, with effects on intestinal inflammation.^59^ Interestingly, studies in mice have also reported beneficial metabolic effects of this mix,^60^ and in humans a protective effect of VSL#3 against increased fat mass has been evidenced in healthy young adults consuming a HF diet.^61^ The mechanism of action was, however, not reported in these studies. Very recently, two studies reported that improvement of Mets parameter in HF-diet fed mice by probiotic supplementation (a mix of *Limosilactobacillus vaginalis* FN3 and *Bifidobacterium animalis* subsp. *lactis* F1–7^62^ and *Lactobacillus plantarum* LP104)^63^ was possibly associated with regulation of BA metabolism and reduce FXR-Fgf15 pathway in the ileum.

Our work presents some limitations despite the fact that we tried to explore most of the pathways and mechanisms classically proposed in the literature to explain the beneficial action of probiotics in rodent models of MetS or obesity. First of all, the amplitudes of the gene expression regulation in the different tissues were quite low and the real involvement of such changes in the metabolic outcomes remains to be demonstrated. Secondly, the bile acid composition was studied in the caecum and, even if the caecum is closely located after the ileum, one could not exclude that additional modifications of bile acid species occurred at the level of the ileum, contributing to the inhibition of the FXR activity. In addition, modifications of the microbiota composition in the small intestine might have contributed more directly to the beneficial effects of the probiotic mix rather than the changes detected in the feces. Finally, although the role of colonization of the gut by probiotics in explaining their health effects is still a matter of debate in the scientific community, the possible mucosal adherence and intestinal colonization of our strains were not studied in the present work.

In conclusion, although there have already been several studies, both in rodents and humans, investigating the potential of *Lactobacillus* and *Bifidobacterium* strains in improving lipid metabolism in MetS and obesity, here we evaluate the efficacy and the mechanisms of action of *B. animalis* subsp. *lacti*s LA804 and *L. gasseri* LA806, two strains carefully selected after a multi-criteria screening^20^. We discovered that the inhibition of FXR signaling in the small intestine could contribute to the beneficial action of these strains, independently from the more classically described protective effects of lactic bacteria on gut permeability, SCFA production, or GLP1 induction. Inhibition of the intestinal FXR/Fgf15 pathway was associated with modulation of the cecal BA profile with increased FXR antagonist/agonist ratio among BA metabolites and subtle changes in gut microbiota composition that may have contributed to this process. Although the translation from animals to humans of the beneficial effects and involved mechanisms remains to be established, the fact that several trials with *Lactobacillus* and *Bifidobacterium* strains have already demonstrated weight and metabolic improvements^14,15,18,61^ positively support *B. animalis* subsp. *lacti*s LA804 and *L. gasseri* LA806 strains as a promising probiotic mix for the management of weight and metabolism in MetS and obesity.

## Materials and methods

### Mouse model, experimental design, and tissue sampling

The study protocol was approved by the local ethics committee with the agreement number CECAPP_LS_2021_006. Forty-eight C57BL/6J male mice were purchased from Envigo, France, at the age of 5 weeks. Animals were housed in an air-conditioned room with a controlled environment of 21 ± 0.5°C and 60–70% humidity with a 12-h light/dark cycle and with free access to food and water. After acclimatization for 1 week, mice were randomly divided into three groups: the Chow diet group, the high-fat diet group (HF), and the high-fat diet + probiotic mix group (HF-Pr2). The Chow diet group received the control Chow diet (LASQC diet® Rod-16 R, LASvendi). Both high-fat diet groups received a diet containing 61% of the energy from fat (292HF-FEDSAFE, composition in Supplementary Table S2) and the HF-Pr2 group was supplemented daily with a mixture of two probiotic strains (detailed below). The nutritional intervention lasted for 12 consecutive weeks. Body weight was measured once a week and food intake monitored three times a week throughout the experiment. At the end of the study, animals were euthanized after a 12-h fasting (Supplementary Figure S1). Blood and tissues were immediately collected, weighed, and treated accordingly to subsequent analysis requirements and then stored at 80°C. Fasting blood glucose levels were measured using a OneTouch glucometer (Johnson&Johnson, New Brunswick, United States).

### Probiotic mix

The probiotic mix was provided by PiLeJe Laboratoire (Paris, France) in the form of a freeze-dried mixture of two probiotic strains (*B. animalis* subsp. *lactis* LA804 and *L. gasseri* LA806) with a ratio of 1:1. PBS was added to the mix in order to administer 1.10^9^ CFU/day to each mouse. HF-Pr2 mice were force-fed daily, every morning, with the probiotic mix from the first day of a high-fat diet until the end of the experiment. Mice from the Chow diet and HF groups received PBS only. To conform to the animal ethics committee’s instructions, probiotics and PBS were administered by intra-oral depot during the first 6 weeks of treatment followed by intragastric gavage until the end of the experiment.

### Measurements of metabolic markers

Liver triglyceride (TG) and total cholesterol concentrations were measured with commercial kits (BIOLABO, ref #80019 and #K1106 respectively). Plasma insulin level was measured by ELISA (Crystal Chem, ref #90080). All assays were performed according to the instructions of the manufacturers.

### Oral lipid tolerance test (OLTT)

Animals fasted for 12 h before OLTT. The test was performed after 10 weeks of treatment and consisted in the administration of 250 µL of Isio4® vegetable oil, a mixture of rapeseed, sunflower, oleic sunflower, and linseed oils with balanced polyunsaturated fatty acid profile (Lesieur). The oil was administered by intragastric gavage. Blood samples were collected from the tail to measure fasting (before the test) and postprandial lipemia (1 h, 2 h, and 4 h post-gavage). Blood was centrifuged to measure plasma triglyceride concentrations using a commercial kit in accordance with the manufacturer’s instructions (BIOLABO ref #80019).

### RNA extraction and real-time quantitative PCR (RT-qPCR)

Total RNA from several tissues (liver, white/brown adipose tissues, and intestinal segments) were extracted using TRI-Reagent (Sigma Aldrich, ref #T9424), in accordance with the manufacturer’s instructions. First-strand cDNAs were synthesized from 1 µg of total RNAs using TAKARA Prime Script™ RT Reagent (TAKARA Bio, ref #RR037A). Purity and concentration of RNAs were determined using NanodropOne (Ozyme, Saint-Cyr-L’Ecole, France), and the quality was checked by using a Bioanalyser (Agilent Technologies, Santa Clara, United-States). Real-time PCR assays were performed with Rotor-Gene Q (Qiagen, Hilden, Germany) using SYBR® Premix Ex-Taq™ (TAKARA Bio, ref #RR420L). TATA-box binding protein (TBP) was used as a reference gene to normalize the results, as previously reported.^64^ Primers are listed in Supplementary Table S3.

### Hepatic lipid droplet analysis

After dissection, the liver was immediately fixed in 4% paraformaldehyde and then paraffin-embedded for the analysis of lipid content. The prepared series of 10-µm sections were stained with Oil Red O (Biognost, ref #ORO-k-250) to determine the accumulation of neutral lipids in the liver. The surface area of the lipid droplets (µm^2^) was quantified per acquisition field using a custom-written Fiji macro.

### 16s RNA gene sequencing and data processing

Analysis of the intestinal microbiota at the end of the experiment was carried out by sequencing bacterial 16S rRNA in feces by the ProfilExpert platform (Lyon, France). Purified DNA were extracted from 15 mg of feces with the Zymobiomics DNA microprep kit (Zymo Research, ref #D4301). 40 ng of DNA were used for the generation of libraries targeting the V3-V4 regions (Quick-16S NGS Library Prep Kit, Ozyme) and then sequenced on Illumina MiSeq standard v3. The sequences were “demultiplexed” using Bcl2fastq software (v2.17.1.14) then cleaned with cutadapt (v1.9.1). Amplicon sequence variants (ASVs) were identified using Qiime2 DADA2 with a Zero noise OTU approach. Finally, a taxonomic assignment was made with Greengenes-13.8-nr99 base. Alpha and beta diversities were calculated under RStudio (v2021.09.1 build 372) using the phyloseq (v1.34.0) and metagenomeseq (1.32.0) libraries.

### Quantification of caecal bile acids

The quantification of bile acids was carried out from 100 mg of frozen caecum by High-Performance Liquid Chromatography coupled with tandem Mass Spectrometry (HPLC-MS/MS) according to the method described by Humbert *et al*.^65^ and as previously reported.^66^ The results are expressed in nmol/g of caecum (wet weight).

### Statistical analysis

Statistical analyses and graphical representations were performed using RStudio (V2021.09.1 Build 372) and GraphPad Prism 9. For each parameter, normality (Shapiro test), homogeneity of variances (Levene test), and independence of residuals (Durbin Watson test) were tested. Depending on the results, either a Kruskal–Wallis test followed by Benjamini–Hochberg (BH) multiple correction and a Dunn posthoc test or a one-way Anova with BH multiple correction followed by a posthoc Tukey test were used to compare groups. Nonparametric Spearman rank correlations between markers of metabolic syndrome, intestinal FXR pathway, and intestinal lipid uptake were conducted using nonparametric Spearman’s test and visualized using a correlation matrix and scatter plots. PLS-DA of the data were performed on microbial data and on the whole set of data generated in the study. Variable Importance of Projection (VIP) scores were assessed to rank parameters for their degree of discrimination within the model. Data are presented as the mean ± SEM. The results were considered significant when *p < 0.05*. Symbols are used to illustrate significant differences with the Chow diet-fed group (^$^*p* < 0.05; ^#^*p* < 0.01 and ^¤^*p* < 0.001) and stars are used to illustrate significant differences between HF-Pr2 and HF groups (*p < 0.05; **p < 0.01 and ***p < 0.001).

## Supplementary Material

Supplemental Material

Supplemental Material

## Data Availability

The data that support the findings of this study are available from the corresponding author upon reasonable request. The data of 16S sequencing have been deposited in the French repository at “Recherche.data.gouv.fr” and can be accessible with the following link: https://doi.org/10.57745/MXOF51.
