# Supplementary material for: Inhibition of intestinal FXR activity as a possible mechanism for the beneficial effects of a probiotic mix supplementation on lipid metabolism alterations and weight gain in mice fed a high fat diet
Source: Gut Microbes. 2023 Nov 20;15(2):2281015. doi: 10.1080/19490976.2023.2281015 (PMC10730200; doi:10.1080/19490976.2023.2281015)
Supplement: Supplemental Material [file KGMI_A_2281015_SM1615.docx]

**Inhibition of intestinal FXR activity as a possible mechanism for the beneficial effects of a probiotic mix supplementation on lipid metabolism alterations and weight gain in mice fed a high fat diet.**

**Supplementary Figures and Tables**

**
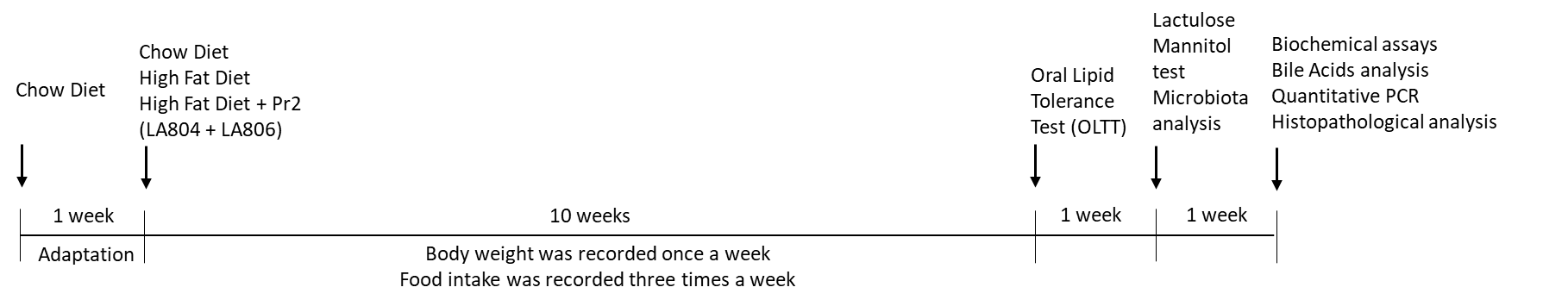
**

**Supplementary Figure 1.** **Study design.** C57BL/6 mice were conditioned for 1 week. For the following 12 weeks, mice were divided into three groups, Chow diet (Chow), High Fat (HF) and HF with probiotics (HF-Pr2). Body weights and food intakes were monitored weekly and three times a week, respectively. At the endpoint (12 weeks), mice were euthanized, and blood and tissue samples were collected (n=12-16).

**Supplementary Figure 2.** **Effects of the probiotic mix on Brown adipose tissue activation, white adipose tissue browning and liver lipid oxidation-related genes.** Relative mRNA expression levels of Uncoupling protein 1 (*Ucp1*), Cell death-inducing DNA fragmentation factor alpha subunit-like effector A (*Cidea*), Peroxisome proliferator-activated receptor gamma coactivator 1 alpha (*Pgc1α*) and PR/SET domain containing 16 (*Prdm16*) in brown adipose tissue. Relative mRNA expression levels of *Pgc1α* and *Prdm16* in eWAT. Relative mRNA expression levels of Carnitine palmitoyltransferase 1 alpha (*Cpt1α*), Peroxisome proliferator activated receptor alpha (*Ppa*r*α*), *Pgc1α*, Pyruvate dehydrogenase kinase isozyme 4 (*Pdk4*), Uncoupling protein 2 (*Ucp2*), Acyl-coenzyme A oxidase 1 palmitoyl (*Acox1*) and Peroxisomal biogenesis factor 2 (*Pex2*) in the liver. Data are mean ± SEM (n=5-10), ^$^ *p < 0.05*, ^#^ *p < 0.01*, ^¤^ *p < 0.001* *versus* the Chow diet group.

**
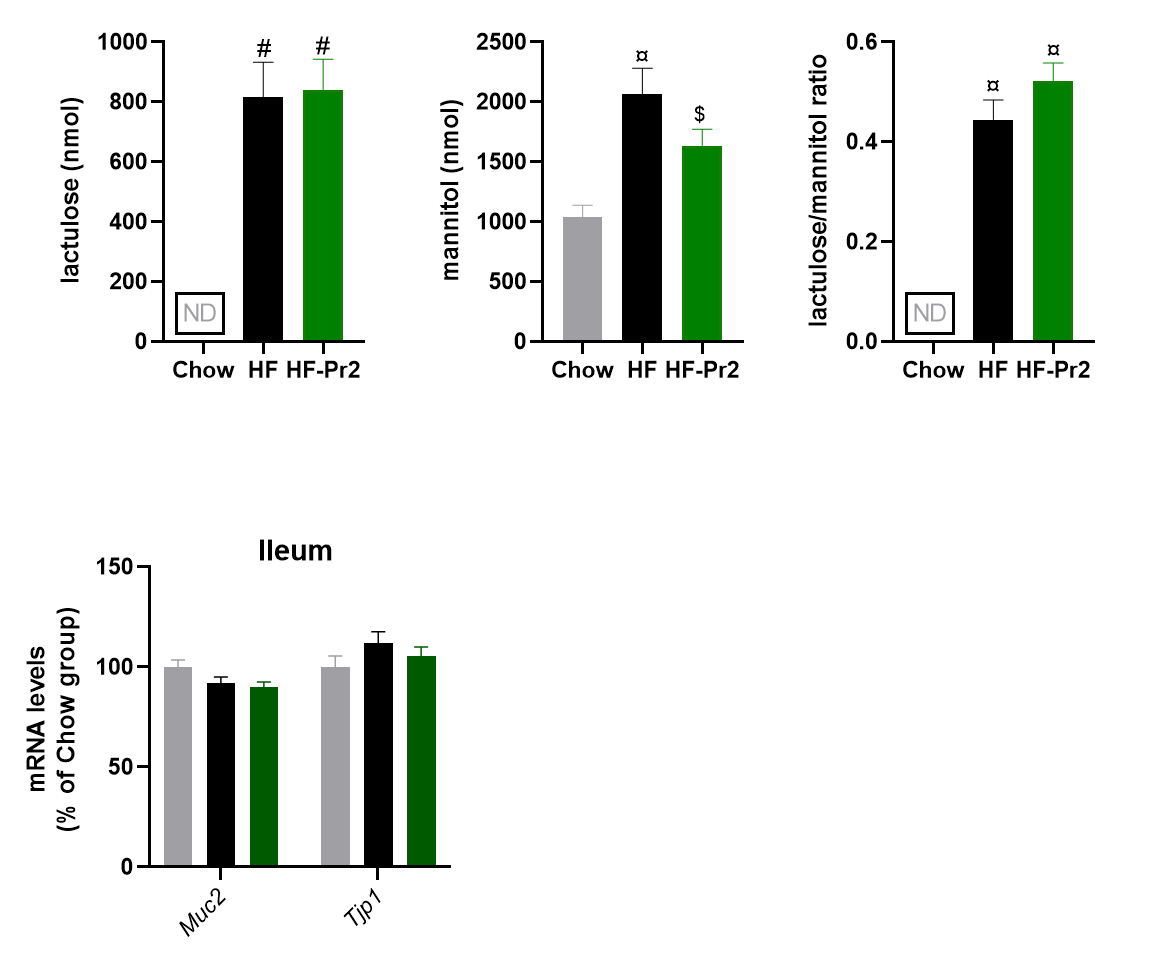
**

**Supplementary Figure 3.** **Intestinal permeability estimated by the lactulose/mannitol** **test.** Urinary lactulose, mannitol and lactulose/mannitol ratio. Data are mean ± SEM (n=7-8), ^$^ *p < 0.05*, ^#^ *p < 0.01*, ^¤^ *p < 0.001* *versus* the Chow diet group. Relative mRNA expression levels of Mucin 2 (*Muc2*) and Tight junction protein 1 (*Tjp1*) in ileum. Data are mean ± SEM (n=7-12).

**Supplementary Figure 4.** **Graphical representation of the 7 ASVs (amplicon sequence variants) increased by supplementation with the probiotic mix.** ^$^ *p < 0.05*, ^#^ *p < 0.01*, ^¤^ *p < 0.001* *versus* the Chow diet group and **p* < 0.05, ***p* < 0.01, ****p* < 0.001 HF-Pr2 *versus* the HF group.

**Supplementary Figure 5.** **Graphical representation of the 10 ASVs (amplicon sequence variants) decreased by supplementation with the probiotic mix.** ^$^ *p < 0.05*, ^#^ *p < 0.01*, ^¤^ *p < 0.001* *versus* the Chow diet group and **p* < 0.05, ***p* < 0.01, ****p* < 0.001 HF-Pr2 *versus* the HF group.

**Supplementary Figure 6.** **Effects of the supplementation with the probiotic mix on short chain fatty acid amounts in the feces.**

Data are mean ± SEM (n=5).


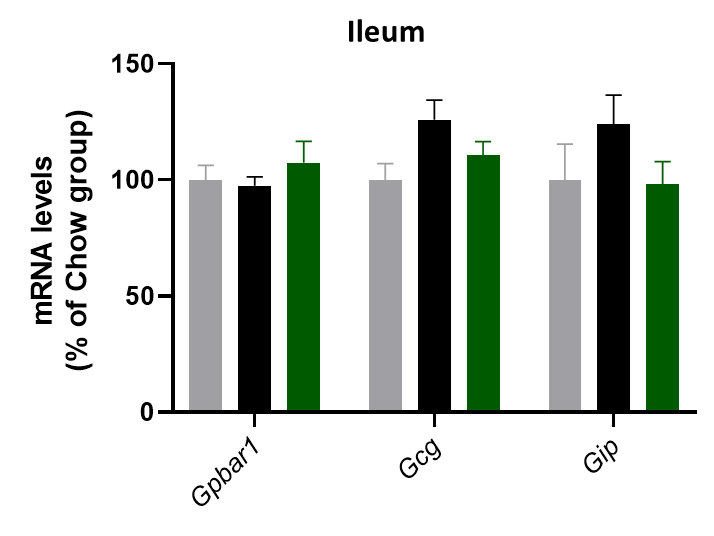


**Supplementary figure 7. Effects of the supplementation with the probiotic mix on Gpbar1, Gcg and Gip gene expressions.**

Relative mRNA expression levels of G protein-coupled bile acid receptor 1 (*Gpbar1*), Glucagon (*Gcg*) and Gastric inhibitory polypeptide (*Gip*) in ileum and jejunum. Data are mean ± SEM (n=7-12), ****p* < 0.001 HF-Pr2 *versus* the HF group and ^¤^ *p < 0.001* *versus* the Chow diet group.


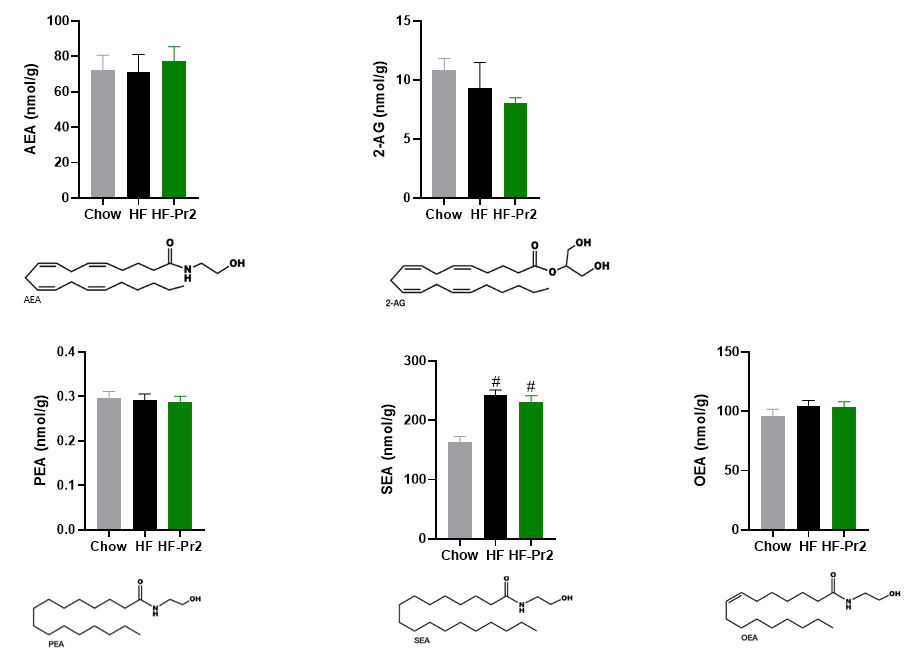


**Supplementary Figure 8. Effects of supplementation with the probiotic mix on endocannabinoid and N-acetylamine concentrations in the small intestine.** AEA (N-arachidonoylethanolamide), 2-AG (2-arachidonoylglycerol), PEA (Palmitoylethanolamide), SEA (stearoylethanolamide) and OEA (Oleoylethanolamide). Data are mean ± SEM (n=7-9), ^#^ *p* < 0.01 *versus* the Chow diet group.

Molecule representations from N.M. Kogan and R. Mechoulam The chemistry of endocannabinoids 2006


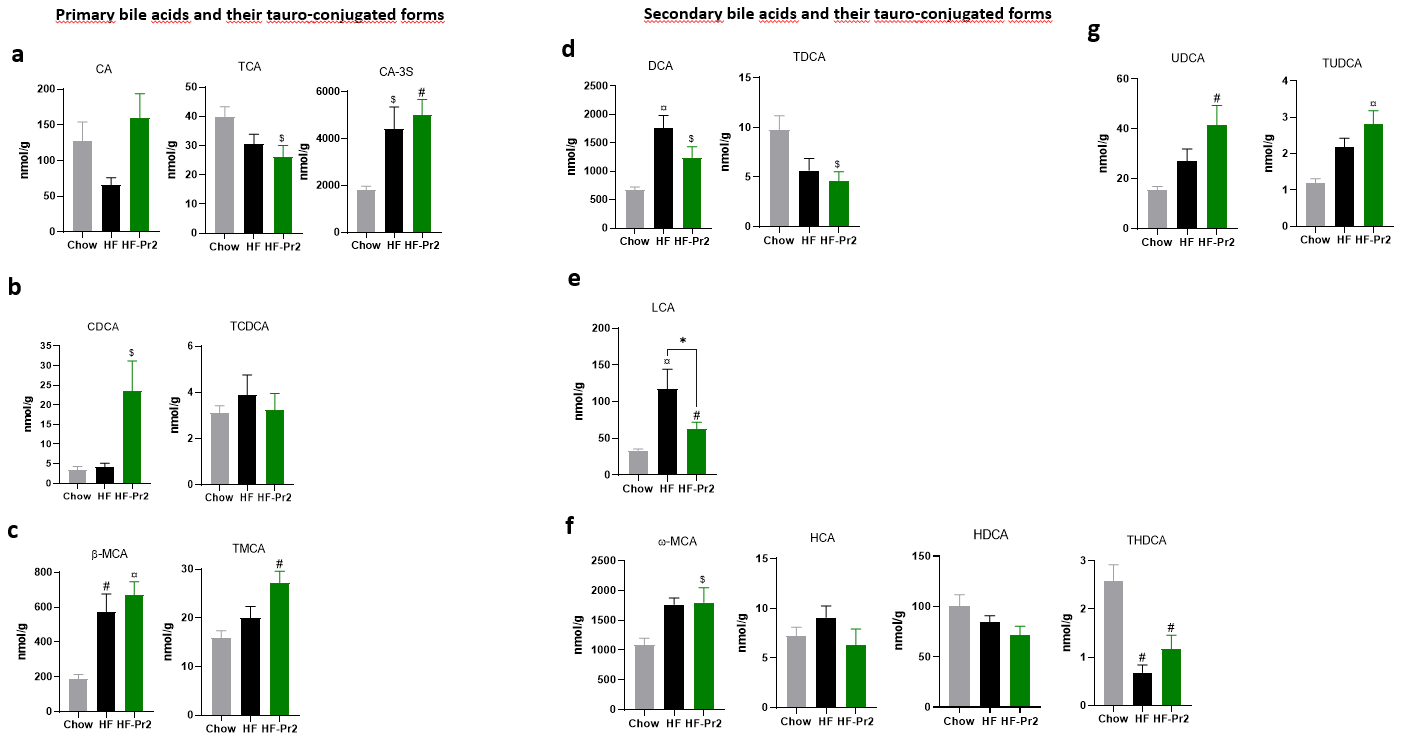


**Supplementary Figure 9. Amounts of primary and secondary bile acids and their tauro-conjugated forms in the caecum.** Primary bile acids **(a)(b)** and **(c)**. Secondary bile acids **(d)(e)** and **(f)**. **(g)** UDCA and TUDCA. Data are mean ± SEM (n=6-10). ^$^ *p < 0.05*, ^#^ *p < 0.01*, ^¤^ *p < 0.001* *versus* the Chow diet group and **p < 0.05* HF-Pr2 *versus* the HF group.

**Supplementary Figure 10. Effects of the probiotic mix on bile acid metabolism-related genes in the liver.** Relative mRNA expression levels of Cytochrome P450 family 7 subfamily a polypeptide 1 (*Cyp7a1*), Cytochrome P450 family 8 subfamily b polypeptide 1 (*Cyp8b1*), Cytochrome P450 family 27 subfamily a polypeptide 1 (*Cyp27a1*), Cytochrome P450 family 7 subfamily b polypeptide 1 (*Cyp7b1*), Nuclear receptor subfamily 1 group H member 4 (*Nr1h4*), Nuclear receptor subfamily 0, group B, member 2 (*Nr0b2*), ATP-binding cassette sub-family B member 11 (*Abcb11*) and Solute carrier family 10 (sodium/bile acid cotransporter family) member 1 (*Slc10a1*) in liver samples (n=7-10). Data are mean ± SEM, ^$^ *p < 0.05*, ^#^ *p < 0.01* *versus* the Chow diet group.


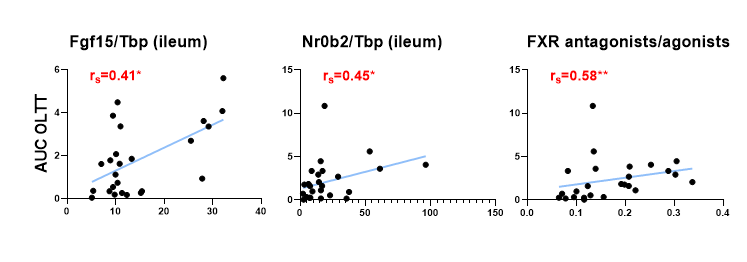


**Supplementary Figure 11.** **Spearman correlations between AUC of the OLTT and inhibition of intestinal FXR pathway.** Data from all groups of animals were used. **p* < 0.05 and ***p* < 0.01.


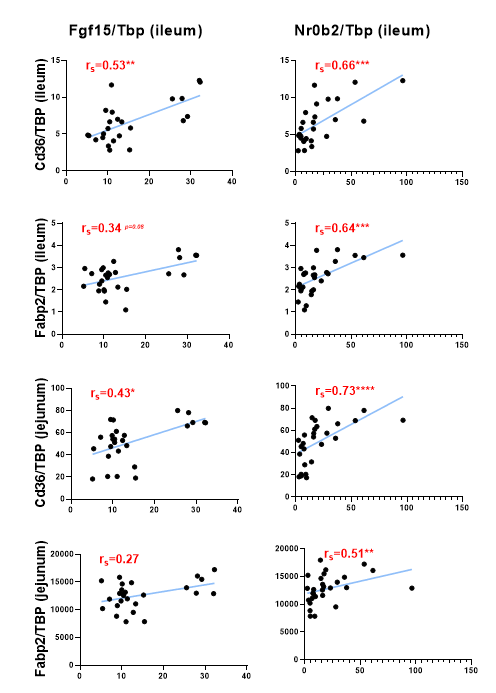


**Supplementary Figure 12.** **Spearman correlations between *Fgf15* and *Nrob2* expression in the ileum and fatty acid uptake-related genes in the ileum and jejunum.** Data from all groups of animals were used. **p* < 0.05, ***p* < 0.01, ****p* < 0.001.


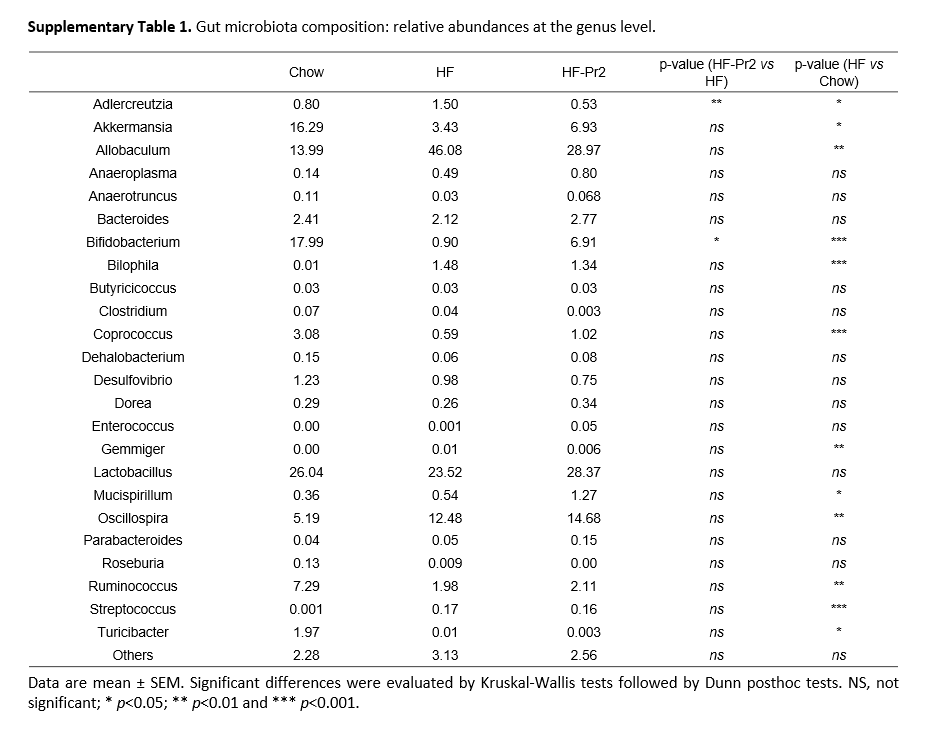


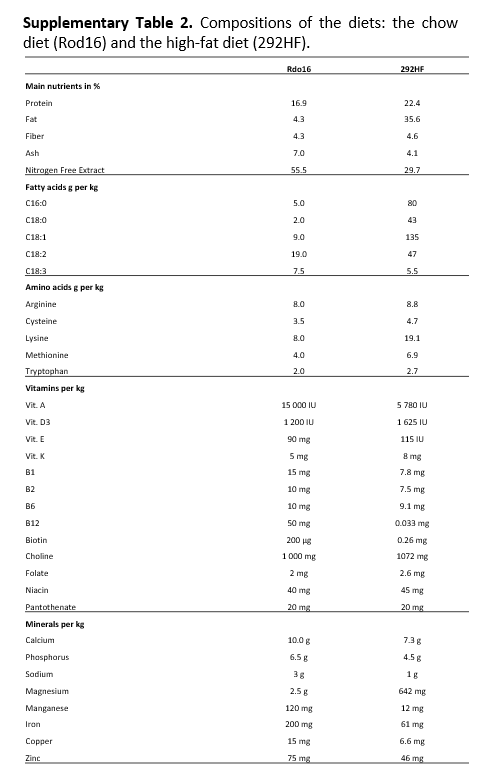


**Supplementary** **Table 3.** Primer sequences

**Supplementary** **Table 4.** mRNA levels (raw values in arbitrary units) for each figure.

|  |  |  | **Chow** | | **HFD** | | **HFD + P2** | |
| --- | --- | --- | --- | --- | --- | --- | --- | --- |
|  |  |  | mean | *SD* | mean | *SD* | mean | *SD* |
| **FIGURE 2** | Fig. 2A (liver) | *LipC* | 25.3 | *4.9* | 36.1 | *11.2* | 24.3 | *2.5* |
|  |  | *Cd36* | 33.9 | *7.3* | 36.5 | *7.9* | 19.7 | *4.1* |
|  |  | *Srebp-1c* | 5.3 | *1.6* | 11.2 | *1.8* | 6.2 | *2.8* |
|  |  | *Fasn* | 2.5 | *0.7* | 3.0 | *0.7* | 2.2 | *0.6* |
|  |  | *Dgat2* | 28.1 | *6.8* | 43.9 | *10.5* | 25.9 | *6.3* |
|  |  |  |  |  |  |  |  |  |
|  | Fig. 2B (eWAT) | *Lpl* | 102.9 | *17.3* | 159.8 | *26.1* | 139.3 | *24.3* |
|  |  | *Ldl-r* | 67.2 | *17.0* | 226.6 | *43.1* | 96.9 | *35.6* |
|  |  | *Cd36* | 11.6 | *1.65* | 16.11 | *1.84* | 14.16 | *1.72* |
|  |  | *Dgat2* | 135.5 | *29.2* | 574.5 | *78.2* | 334.1 | *134.3* |
|  |  | *Hsl* | 112.4 | *10.8* | 95.9 | *14.5* | 123.1 | *15.1* |
|  |  | *Atgl* | 249.5 | *27.5* | 139.5 | *16.2* | 203.3 | *45.3* |
|  |  |  |  |  |  |  |  |  |
|  | Fig. 2C (liver) | *Gpx1* | 457.7 | *80.2* | 616.8 | *157.3* | 449.5 | *49.0* |
|  |  | *Gpx4* | 34.5 | *6.7* | 33.0 | *6.1* | 23.2 | *2.2* |
|  |  | *Cat* | 460.1 | *60.1* | 597.2 | *157.4* | 405.9 | *45.9* |
|  |  | *Prdx3* | 35.1 | *6.1* | 38.0 | *7.9* | 28.1 | *2.0* |
|  |  | *Sod1* | 80.5 | *13.0* | 84.5 | *16.6* | 69.3 | *6.2* |
|  |  | *Sod2* | 93.4 | *15.0* | 105.3 | *19.5* | 74.8 | *7.3* |
|  |  | *Gsta4* | 37.3 | *7.3* | 32.4 | *8.6* | 22.1 | *2.5* |
|  |  | *Hspa5* | 105.4 | *28.6* | 207.7 | *94.3* | 119.3 | *27.2* |
|  |  | *Atf4* | 28.4 | *4.2* | 36.2 | *9.5* | 27.6 | *4.2* |
|  |  | *Ddit3* | 0.48 | *0.13* | 1.05 | *0.37* | 0.72 | *0.22* |
|  |  |  |  |  |  |  |  |  |
|  | Fig. 2D (eWAT) | *Tnf alpha* | 0.21 | *0.07* | 0.80 | *0.24* | 0.35 | *0.16* |
|  |  | *Ccl2* | 0.53 | *0.19* | 5.30 | *1.17* | 2.01 | *0.51* |
|  |  | *Il-6* | 0.037 | *0.011* | 0.047 | *0.006* | 0.057 | *0.014* |
|  |  | *Lcn2* | 78.0 | *33.4* | 147.6 | *79.9* | 57.2 | *12.3* |
|  |  | *Adipoq* | 14.8 | *2.2* | 13.4 | *3.8* | 16.8 | *2.9* |
|  |  | *Lep* | 0.42 | *0.17* | 2.60 | *0.31* | 0.97 | *0.48* |

|  |  |  | **Chow** | | **HFD** | | **HFD + P2** | |
| --- | --- | --- | --- | --- | --- | --- | --- | --- |
|  |  |  | mean | *SD* | mean | *SD* | mean | *SD* |
| **FIGURE 3** | Fig. 3C (jejunum) | *Cd36* | 38.4 | *16.2* | 70.8 | *6.1* | 50.8 | *16.6* |
|  |  | *Fabp2* | 11681 | *2266* | 15006 | *1699* | 12955 | *2842* |
|  |  | *Dgat1* | 15.8 | *1.1* | 18.6 | *1.4* | 16.2 | *2.0* |
|  |  | *Mttp* | 16.4 | *1.4* | 19.9 | *2.4* | 18.4 | *2.3* |
|  |  | *ApoB* | 13517 | *2923* | 13375 | *2710* | 12318 | *2354* |
|  |  | *Surf4* | 16.9 | *3.6* | 19.5 | *4.8* | 18.2 | *4.4* |
|  |  |  |  |  |  |  |  |  |
|  | Fig. 3D (ileum) | *Cd36* | 5.0 | *1.8* | 9.6 | *2.1* | 5.8 | *1.7* |
|  |  | *Fabp2* | 2.3 | *0.7* | 3.4 | *0.5* | 2.4 | *0.4* |
|  |  | *Dgat1* | 20.0 | *5.7* | 25.2 | *3.8* | 21.4 | *4.6* |
|  |  | *Mttp* | 22.4 | *5.4* | 24.1 | *2.5* | 20.6 | *4.3* |
|  |  | *ApoB* | 26.0 | *8.9* | 25.8 | *5.9* | 23.9 | *9.0* |
|  |  | *Surf4* | 24.3 | *8.0* | 24.3 | *3.8* | 23.8 | *5.6* |

|  |  |  | **Chow** | | **HFD** | | **HFD + P2** | |
| --- | --- | --- | --- | --- | --- | --- | --- | --- |
|  |  |  | mean | *SD* | mean | *SD* | mean | *SD* |
| **FIGURE 5** | Fig. 5A (ileum) | *Gpbar1* | 63.8 | *14.0* | 62.2 | *6.4* | 68.5 | *18.8* |
|  |  | *Gcg* | 18.2 | *4.3* | 23.0 | *4.1* | 20.2 | *3.5* |
|  |  | *Gip* | 20.7 | *11.1* | 25.6 | *7.0* | 20.3 | *6.4* |
|  |  |  |  |  |  |  |  |  |
|  | Fig. 5B (ileum) | *Napepld* | 17.7 | *2.3* | 21.8 | *4.1* | 22.1 | *2.8* |
|  |  | *Naaa* | 21.7 | *4.4* | 25.9 | *2.4* | 21.2 | *4.1* |
|  |  | *Faah* | 14.5 | *4.8* | 23.0 | *3.3* | 18.7 | *5.4* |
|  |  |  |  |  |  |  |  |  |
|  | Fig. 5C (ileum) | *Ahrr* | 62.6 | *15.0* | 46.7 | *10.8* | 61.3 | *19.2* |
|  |  | *Cyp1a1* | 95.5 | *29.4* | 51.3 | *21.5* | 98.5 | *76.1* |
|  |  | *Cyp1b1* | 68.6 | *12.1* | 52.2 | *2.5* | 66.5 | *23.8* |
|  |  |  |  |  |  |  |  |  |
|  | Fig. 5D (ileum) | *Nr1h4* | 23.3 | *4.5* | 18.5 | *2.1* | 20.8 | *4.3* |
|  |  | *Nr0b2* | 12.2 | *11.0* | 44.8 | *28.1* | 11.5 | *5.0* |
|  |  | *Fgf15* | 10.5 | *3.4* | 29.1 | *2.6* | 10.1 | *1.7* |

|  |  |  | **Chow** | | **HFD** | | **HFD + P2** | |
| --- | --- | --- | --- | --- | --- | --- | --- | --- |
|  |  |  | mean | *SD* | mean | *SD* | mean | *SD* |
| **Supp FIG 2** | Brow adipose tissue | *Ucp1* | 38.6 | *14.1* | 71.7 | *22.0* | 66.3 | *15.5* |
|  |  | *Cidea* | 119.3 | *21.2* | 113.6 | *13.4* | 117.1 | *32.0* |
|  |  | *Pgc1 alpha* | 0.35 | *0.12* | 0.44 | *0.10* | 0.34 | *0.06* |
|  |  | *Prdm16* | 177.6 | *52.5* | 251.3 | *56.4* | 200.6 | *43.0* |
|  |  |  |  |  |  |  |  |  |
|  | eWAT | *Pgc1 alpha* | 0.042 | *0.009* | 0.021 | *0.005* | 0.024 | *0.003* |
|  |  | *Prdm16* | 123.0 | *36.4* | 217.1 | *52.1* | 174.6 | *31.0* |
|  |  |  |  |  |  |  |  |  |
|  | Liver | *Cpt1 alpha* | 38.7 | *6.1* | 28.5 | *6.5* | 26.7 | *3.3* |
|  |  | *Ppar alpha* | 32.5 | *7.6* | 35.3 | *10.9* | 27.8 | *6.0* |
|  |  | *Pgc1 alpha* | 0.12 | *0.03* | 0.07 | *0.01* | 0.05 | *0.01* |
|  |  | *Pdk4* | 1.7 | *0.9* | 1.1 | *0.6* | 1.2 | *0.6* |
|  |  | *Ucp2* | 25.8 | *3.3* | 40.8 | *10.8* | 31.7 | *8.2* |
|  |  | *Acox1* | 100.0 | *16.4* | 121.4 | *42.9* | 85.5 | *20.7* |
|  |  | *Pex2* | 374.2 | *74.7* | 433.0 | *122.9* | 353.7 | *74.9* |

|  |  |  | **Chow** | | **HFD** | | **HFD + P2** | |
| --- | --- | --- | --- | --- | --- | --- | --- | --- |
|  |  |  | mean | *SD* | mean | *SD* | mean | *SD* |
| **Supp FIG 3** | Ileum | *Muc2* | 248.1 | *30.8* | 227.8 | *21.1* | 222.7 | *22.2* |
|  |  | *Tjp1* | 2.3 | *0.4* | 2.5 | *0.3* | 2.4 | *0.3* |

|  |  |  | **Chow** | | **HFD** | | **HFD + P2** | |
| --- | --- | --- | --- | --- | --- | --- | --- | --- |
|  |  |  | mean | *SD* | mean | *SD* | mean | *SD* |
| **Supp FIG 7** | Ileum | *Gpbar1* | 63.8 | *14.0* | 62.2 | *6.4* | 68.5 | *18.8* |
|  |  | *Gcg* | 18.2 | *4.3* | 23.0 | *4.1* | 20.2 | *3.5* |
|  |  | *Gip* | 20.7 | *11.1* | 25.6 | *7.0* | 20.3 | *6.4* |
|  |  |  |  |  |  |  |  |  |
|  | Jejunum | *Gpbar1* | 49.2 | *9.9* | 50.7 | *11.8* | 51.6 | *5.3* |
|  |  | *Gcg* | 17.6 | *2.3* | 24.7 | *3.3* | 22.1 | *2.1* |
|  |  | *Gip* | 159.4 | *21.1* | 265.1 | *28.8* | 194.4 | *48.1* |

|  |  |  | **Chow** | | **HFD** | | **HFD + P2** | |
| --- | --- | --- | --- | --- | --- | --- | --- | --- |
|  |  |  | mean | *SD* | mean | *SD* | mean | *SD* |
| **Supp FIG 10** | Liver | *Cyp7a1* | 30.3 | *20.3* | 30.8 | *9.8* | 31.8 | *11.1* |
|  |  | *Cyp8b1* | 10.9 | *11.0* | 29.5 | *12.4* | 27.3 | *7.9* |
|  |  | *Cyp27a1* | 31.0 | *3.1* | 32.4 | *9.6* | 25.2 | *2.4* |
|  |  | *Cyp7b1* | 46.5 | *13.7* | 103.9 | *45.2* | 95.6 | *39.1* |
|  |  | *Nr1h4* | 27.9 | *5.7* | 28.0 | *6.4* | 22.2 | *1.9* |
|  |  | *Nr0b2* | 29.8 | *17.6* | 34.4 | *18.0* | 30.3 | *8.3* |
|  |  | *Abcb11* | 27.0 | *5.3* | 36.7 | *13.2* | 26.1 | *3.0* |
|  |  | *Slc10a1* | 30.6 | *5.4* | 32.2 | *7.6* | 25.1 | *3.0* |
