# Supplementary material for: Inhibition of intestinal FXR activity as a possible mechanism for the beneficial effects of a probiotic mix supplementation on lipid metabolism alterations and weight gain in mice fed a high fat diet
Source: Gut Microbes. 2023 Nov 20;15(2):2281015. doi: 10.1080/19490976.2023.2281015 (PMC10730200; doi:10.1080/19490976.2023.2281015)
Supplement: Supplemental Material [file KGMI_A_2281015_SM1613.docx]

**Inhibition of intestinal FXR activity as a possible mechanism for the beneficial effects of a probiotic mix supplementation on lipid metabolism alterations and weight gain in mice fed a high fat diet.**

**Supplemental Methods**

***Estimation of intestinal permeability***

A lactulose/mannitol test adapted by Arrieta *et al.^1^* was performed after 11 weeks of treatment to evaluate intestinal permeability. Mice were fasted for 4 hours before receiving a subcutaneous injection of 500 μL NaCl (0.5°/ₒₒ) to increase urine production. Half an hour later, they were force-fed with 200 μL of lactulose (60 mg/mL) and mannitol (40 mg/mL) (Sigma Aldrich, Saint-Louis, United-States). The urine produced throughout the four hours following gavage was collected, centrifuged, and then stored. Concentrations of lactulose and mannitol were measured by colorimetric assays (EnzyChrom^TM^, Clinisciences, Nanterre, France).

***Quantification of short chain fatty acids in the feces***

Feces were frozen in liquid nitrogen and stored at −80 °C. Then samples were crushed in liquid nitrogen and the powder (about 100 mg) precisely weighed. All following steps were done at +4 °C. 2-ethylbutyric acid was spiked in each sample as internal standard. Samples were suspended in HCl 18 mM, vigorously vortexed using zirconium oxide beads (diameter 2.8mm, OZYME, Saint Cyr l'Ecole, France) and then centrifuged at 4000 *g* for 30 min. The pH of the supernatant was adjusted between 2 and 3 (< SCFA pKa) using HCl 1N and was filtered (Chromafil Xtra CA-45/13- cellulose acetate, Macherey Nagel, Duren, Germany) before analysis.

Feces SCFA concentrations were determined by gas chromatography - mass spectrometry (GC-MS) in electron impact ionization (GC-EI/MS, 5977B, Agilent, Massy, France). Briefly, the sample (1µL) was injected automatically into the gas chromatograph (model GC8890, Agilent Technologies), in the split mode (split ratio 5:1), in a fused-silica column (30m x 0.25mm x 0.25µm, DB-FFAP; Agilent, Massy, France). The injector was kept at 250°C and the oven at 80°C for 1 min. The SCFAs were separated at constant flow (1.2 mL.min^-1^) with the following oven program: (a) 80 °C; (b) increase at a rate of 20 °C.min^-1^ to 120 °C; (c) increase at a rate of 10 °C.min^-1^ to 205 °C and hold for 2.5 min. A post run of 1 min at 240°C was applied between each injection. Mass spectrometry acquisition was performed in SIM/SCAN mode. Specific ions from the selected ion monitored signal were used for the SCFA quantification (m/z 60 for acetate, butyrate and valerate; m/z 74 for propionate). A 7-point calibration curve was prepared with a standard solution (Volatil Free Acid mix, 10 mM, Supelco) in HCl 3.5 mM. The data were expressed in µg/mg of fresh feces.

***Quantification of endocannabinoids and N-acetylamines in intestinal tissue***

Anandamide (AEA), 2-arachidonoylglycerol (2-AG) and other N-acetylamines (PEA, OEA, SEA) in ileum samples were measured at the MASSMET platform of Louvain Catholic University (Brussels, Belgium) operated by prof. G. G. Muccioli (<https://uclouvain.be/en/technology-platforms/massmet.html>), by HPLC-MS using a LTQ Orbitrap mass spectrometer (ThermoFisher Scientific, Merelbeke, Belgium) coupled to an Accela HPLC system (ThermoFisher Scientific). The methodology has been described^2^, and the data were normalized by tissue sample weight.

**References**

1. Arrieta, M.C., Madsen, K., Doyle, J., and Meddings, J. (2009). Reducing small intestinal permeability attenuates colitis in the IL10 gene-deficient mouse. Gut *58*, 41–48. 10.1136/gut.2008.150888.

2. Muccioli, G.G., Naslain, D., Bäckhed, F., Reigstad, C.S., Lambert, D.M., Delzenne, N.M., and Cani, P.D. (2010). The endocannabinoid system links gut microbiota to adipogenesis. Mol. Syst. Biol. *6*, 392. 10.1038/msb.2010.46.
